# Pharmacist-Led Management Model and Medication Adherence Among Patients With Chronic Heart Failure: A Randomized Clinical Trial

**DOI:** 10.1001/jamanetworkopen.2024.53976

**Published:** 2024-12-20

**Authors:** Lingjiao Wang, Yuanyuan Zhao, Liping Han, Huan Zhang, Hejun Chen, Aixia Liu, Jing Yu, Ran Fu, Liguang Duan, Feiyue An, Zhimin Guo, Yang Lun, Chaoli Chen, Fangfang Cheng, Chaohui Song, Haixia Gao, Chunhua Zhou

**Affiliations:** 1 Department of Clinical Pharmacy, The First Hospital of Hebei Medical University, Hebei, China; 2 Department of Clinical Psychology, Key Laboratory for Neuroimmunological Regulation and Mental Health of Hebei Province, Hebei, China; 3 Department of Clinical Psychology, The First Hospital of Hebei Medical University, Hebei, China; 4 Department of Clinical Pharmacy, Handan First Hospital, Hebei, China; 5 Department of Pharmacy, Cangzhou Central Hospital, Hebei, China; 6 Department of Pharmacy, Hengshui People’s Hospital, Hebei, China; 7 Department of Pharmacy, the Fourth Hospital of Handan, Hebei, China; 8 Department of Clinical Pharmacy, The Technology Innovation Center for Artificial Intelligence in Clinical Pharmacy of Hebei Province, Hebei, China; 9 Department of Pharmacy, Kanghui Hospital, Tianjin, China; 10 Department of Pharmacology, Center for Innovative Drug Research and Evaluation, Institute of Medical Science and Health, The Hebei Collaboration Innovation Center for Mechanism, Diagnosis and Treatment of Neurological and Psychiatric Disease, The Key Laboratory of Neural and Vascular Biology, Ministry of Education, Hebei Medical University, Hebei, China

## Abstract

**Importance:**

Poor medication adherence is associated with high morbidity and mortality among patients with chronic heart failure (CHF), which is particularly concerning in China.

**Objective:**

To assess the effect of a pharmacist-led management model incorporating a social media platform vs usual care on medication adherence in patients with CHF.

**Design, Setting, and Participants:**

This prospective, multicenter randomized clinical trial was conducted from March 2021 to May 2023, with a follow-up duration of 52 weeks. The trial was conducted in the cardiology wards of 5 hospitals in China. Participants were 18 years or older, had a CHF diagnosis, and were receiving stable medication. They were randomly assigned to either the intervention group (pharmacist-led management) or the control group (usual care) in a 1:1 ratio using a computer-generated random number table with concealed allocation via opaque envelopes. Intention-to-treat data analysis was performed from June 2023 to July 2024.

**Intervention:**

The intervention group received a multimodal pharmaceutical intervention, including WeChat application–based communication and education, and a standardized follow-up visit from a pharmacist every month. The control group received the standardized follow-up visit from nurses every month.

**Main Outcomes and Measures:**

The primary outcome was the proportion of days covered (PDC) by heart failure medication at 52 weeks.

**Results:**

Among the 445 participants analyzed, 223 were assigned to the intervention group and 222 to the control group. These patients had a mean (SD) age of 63.2 (13.3) years and included 263 males (59.1%). A total of 333 patients (74.8%) had a New York Heart Association class III or IV heart failure, indicating severe limitations in physical activity. At 52 weeks, the intervention group had a significantly higher PDC for heart failure medication (8.1%; 95% CI, 5.5%-10.7%; *P* < .001) and a greater proportion of patients with PDC of 80% or greater (odds ratio, 2.94; 95% CI, 1.85-4.67; *P* < .001) compared with the control group.

**Conclusions and Relevance:**

This randomized clinical trial found a modest improvement in medication adherence among patients with CHF who received the pharmacist-led management intervention vs usual care.

**Trial Registration:**

Chinese Clinical Trial Registry Identifier: ChiCTR2000040232

## Key Points

**Question:**

Can a pharmacist-led management model improve medication adherence among patients with chronic heart failure (CHF) compared with a conventional management model?

**Findings:**

In this randomized clinical trial involving 445 patients with CHF in China, patients who were assigned to a pharmacist-led management intervention showed modest improvement in medication adherence at 52 weeks compared with patients assigned to usual care.

**Meaning:**

The findings indicate that a pharmacist-led management model is effective for improving medication adherence among patients with CHF.

## Teaser

This randomized clinical trial examines the effects of a pharmacist-led medication management intervention with a social media component on improving medication adherence in adults with chronic heart failure in China.

## Introduction

Chronic heart failure (CHF) is a complex clinical syndrome that affects approximately 37.7 million people and is a leading cause of morbidity and mortality worldwide.^1,2,3^ There are approximately 13.7 million adults with CHF in China.^4^ The standardized prevalence rates of heart failure are 0.57%, 3.86%, and 7.55% among individuals aged 25 to 64 years, 65 to 79 years, and 80 years or older, respectively.^5^ The case fatality rate of hospitalized patients with heart failure is approximately 4.1%.^6^ Thus, CHF places a heavy burden on individuals, families, and the health system.^4,5,6,7^ Guideline-directed medical therapy is the preferred and most effective way to reduce morbidity and mortality in patients with CHF. This treatment can improve the ejection fraction and quality of life.^8^

Poor medication adherence is an important factor in poor outcomes (eg, rehospitalization) in patients with CHF.^9,10,11,12^ In addition to needing guideline-recommended medications, patients with CHF often need medications for comorbidities and complications, increasing the risk of drug-related problems, such as drug interactions, adverse drug reactions, and poor compliance.^13,14,15^ A number of pharmacist-led or drug-based intervention studies on patients with CHF have shown that clinical pharmacists improve medication compliance, efficacy, and safety.^16,17,18,19^ To our knowledge, no studies have been reported on the effects of the pharmacist-led management model on medication adherence and efficacy among Chinese patients with CHF.

Instant messaging applications have gained popularity, with one such application in China reporting more than 1.2 billion monthly active users.^20^ This popular application is characterized by timeliness and convenience. It is simple to use and thus is not limited by age or cognitive behavior, making it suitable for health interventions in China.^21,22,23,24^ This messaging application has been used in studies on disease and health management in patients with chronic conditions, including AIDS and coronary heart disease, and has demonstrated good results. However, the its effectiveness in the management of patients with CHF needs to be verified.^25,26,27^ Therefore, we conducted a randomized clinical trial to assess the effect of a pharmacist-led management model incorporating a social media platform vs usual care on medication adherence in patients with CHF.

## Methods

### Design and Participants

This prospective, multicenter randomized clinical trial was conducted from March 2021 to May 2023 at the First Hospital of Hebei Medical University, the First Hospital of Handan, Cangzhou Central Hospital, the Fourth Hospital of Handan, and Hengshui People’s Hospital in China. The study was approved by the corresponding ethics committees at these hospitals. All participants provided written informed consent. We adhered to the Consolidated Standards of Reporting Trials (CONSORT) reporting guideline. The trial protocol and statistical analysis plan are provided in Supplement 1.

We included patients with CHF who were admitted to one of the hospitals between March 2021 and April 2022. Inclusion criteria were (1) age 18 years or older; (2) a confirmed CHF diagnosis based on heart failure symptoms and signs, echocardiographic evidence of cardiac structural or functional abnormalities, and increased natriuretic peptide levels; (3) a stable heart failure medication regimen, indicating no acute heart failure exacerbation or medication adjustments in the week before study enrollment; and (4) the ability to understand and provide signed informed consent.

### Procedures

**Figure 1.**
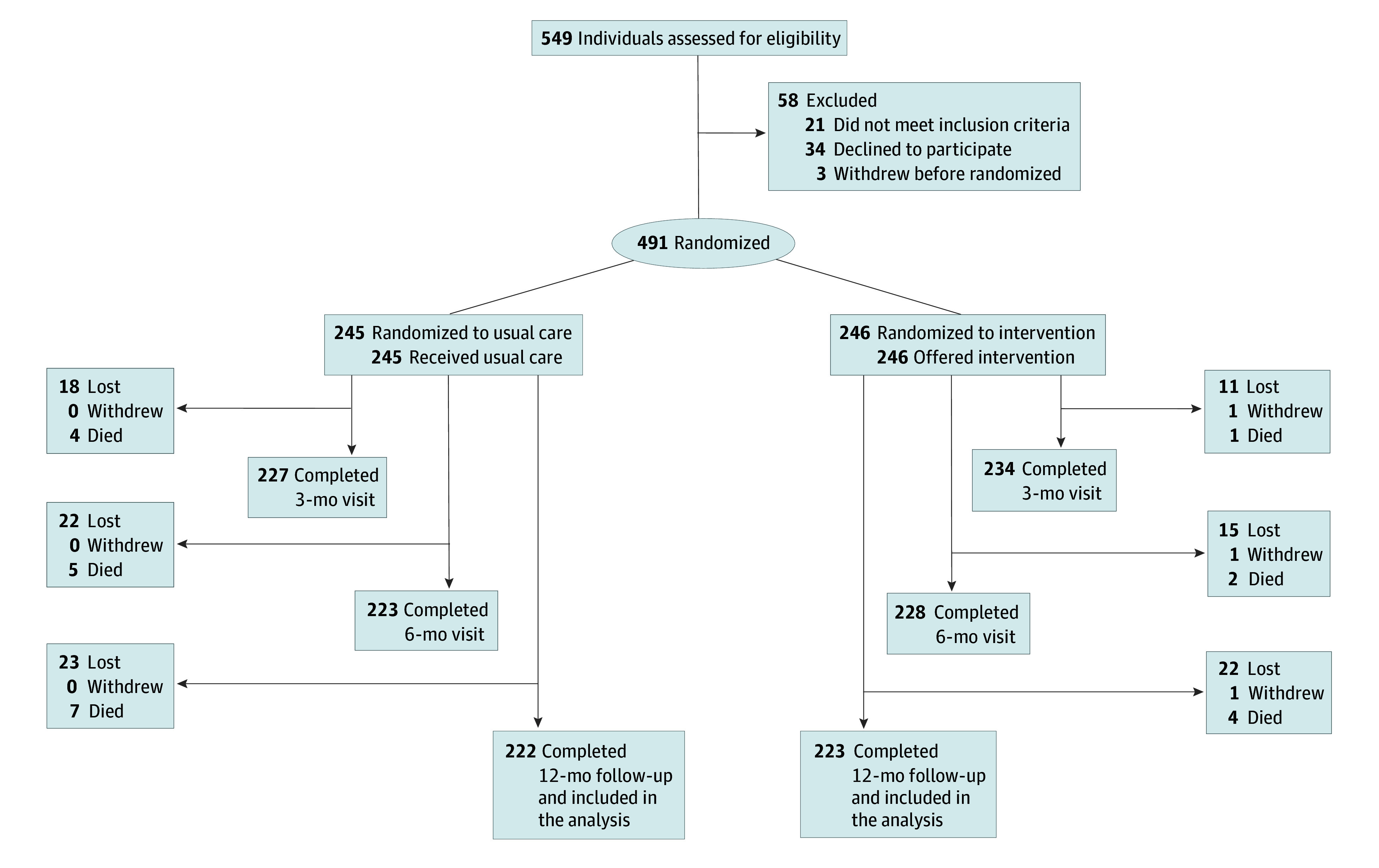
Flow Diagram of Study Participants A total of 445 participants were allocated for analysis.

After collecting baseline data, we categorized eligible patients with CHF into 3 groups based on left ventricular ejection fraction (LVEF): reduced LVEF (<40%), midrange LVEF (40%-49%), and preserved LVEF (≥50%). Following stratification, patients were randomly assigned to the intervention group (pharmacist-led management) or the control group (usual care) in a 1:1 ratio (Figure 1). The randomization process used a computer-generated random number table and was concealed using opaque envelopes. Although the study was open label for researchers and patients, assessors were blinded to the treatment assignments.

#### Usual Care and Intervention 

Patients assigned to the control group underwent follow-up by nurses. The attending physicians prescribed relevant tests based on each patient’s clinical symptoms, adjusted the medication regimen, and issued a medication prescription for 4 weeks (plus or minus 7 days). Nurses reviewed the patients’ medication diaries, compared the diaries with the prescriptions, and inquired about any adverse events and new medications. The nurses instructed the patients to record each medication taken in their medication diary and scheduled the next follow-up visit.

For patients assigned to the intervention group, pharmacists conducted follow-up visits in accordance with the standardized procedures used by nurses for the control group along with the following additional components. Pharmacists (1) added the patient or their caregiver as a WeChat (Tencent Holdings Ltd) friend and created a group in the messaging application; (2) reminded patients to take their medication weekly via this application and responded to medication or disease-related inquiries; (3) provided patient education based on medication adherence, including introduction to misused, missed, newly added, or adjusted medications and information on the drug’s effects, necessity, dose and administration, possible adverse events, handling methods, and drug interactions; and (4) discussed any necessary adjustments to the medication regimen with the attending physician.

If a patient was hospitalized during the follow-up period, the management for both the intervention and control groups was the same. The attending physician determined whether the hospitalization was related to heart failure or other adverse events and continued with follow-up visits according to the standardized procedures.

### Outcomes

Follow-up results were evaluated over a period of 52 weeks. The primary end point was the proportion of days covered (PDC) for heart failure medications at 52 weeks. The secondary end points were the proportion of patients with a PDC of 80% or greater at 52 weeks for heart failure medications and for 4 major drug classes: β-blockers, diuretics, angiotensin-converting enzyme inhibitors or angiotensin receptor blockers (ACEIs or ARBs), and mineralocorticoid receptor antagonists (MRAs). We further analyzed the Morisky score (range: 0-8, with the highest score indicating the highest medication adherence), the Minnesota Living with Heart Failure Questionnaire (MLHFQ) score (range: 0-105, with the highest score indicating lowest quality of life), readmissions, composite end points of all-cause mortality, and days lost due to unplanned cardiovascular hospitalizations or death. The primary safety end point was the incidence of adverse drug events (ADEs).

Participants were thoroughly questioned about ADEs during each follow-up visit. Nurses and pharmacists collected information on ADEs from various sources, including verbal reports from patients or their families, log card records, medical records, and the national adverse drug reaction database, and then recorded this information in the data collection system for assessment by our research team. All recording and assessments of ADEs were conducted according to the standardized procedures for drug adverse reaction monitoring established by the State Administration for Market Regulation of China. Death data were obtained from hospital medical records, death certificates, household cancellation certificates, or cremation certificates provided by families as well as from the Chinese Centers for Disease Control and Prevention.

At the outset of the trial, we established a Data Management Committee composed of experts in the fields of pharmacy, clinical practice, and statistics. At the midpoint and conclusion of the trial, the Data Management Committee conducted a quality control audit of the data related to ADEs, mortality cases, and pharmacist interventions.

### Sample Size Calculation

In a previous study,^16^ the overall SD of PDC for heart failure medications at 52 weeks was 14.9%. Assuming a mean (SD) difference of 4.0 (0.2) between the intervention and control groups and assuming α = .05 and β = 0.20, respectively, 218 cases were required per group. Assuming an estimated attrition rate of 10%, each group required 243 cases, for a total of 486 cases.

### Statistical Analysis

To assess the balance between the control and intervention groups after randomization, we descriptively compared the baseline characteristics using 2-sample *t* tests, χ^2^ tests, Fisher exact tests, or Wilcoxon rank sum tests. Normally distributed continuous variables were described with the mean (SD), whereas nonnormally distributed variables were described using the median (IQR). Categorical variables were described using counts (percentages). To compare results between the intervention and control groups, independent-sample *t* tests or Wilcoxon rank sum tests were used for continuous variables. To control the type I error that may be introduced by multiple comparisons, we applied the Bonferroni correction method to all secondary end points. For categorical variables, χ^2^ tests or exact probability tests were used. Nelson-Aalen curves were plotted to assess the composite end point of heart failure hospitalization and mortality; differences between groups were compared using a log-rank test. *P* < .05 indicated statistical significance.

Subgroup analyses of the primary and secondary efficacy end points were performed to assess the consistency of intervention effects across the following subgroups: age (<75 vs ≥75 years), sex, heart rate at baseline (≤75 vs >75 bpm), New York Heart Association (NYHA) class (I or II [indicating mild limitations in physical activity] vs III or IV [indicating severe limitations in physical activity]), LVEF (<40% vs ≥40%), history of diabetes, level of disease burden (number of different medications at baseline), baseline adherence, and quality of life at baseline.

Intention-to-treat data analysis was performed from June 2023 to July 2024. All analyses used R, version 4.0.3 (R Project for Statistical Computing).

## Results

Figure 1 depicts the flow of participants through the trial. A total of 549 patients were recruited, of whom 491 met the inclusion criteria and were randomized. After withdrawals, deaths, and loss to follow-up, 445 participants were analyzed, of whom 223 were assigned to the intervention group (received pharmacist-led management) and 222 to the control group (received usual care). All baseline characteristics were well balanced between the 2 groups (Table 1). Patients had a mean (SD) age of 63.2 (13.3) years and included 263 males (59.1%) and 182 females (40.9%). Among these patients, 333 (74.8%) had an NYHA class III or IV heart failure. Patients with heart failure often have multiple comorbidities. In this study, 246 patients (55.3%) had hypertension and 202 patients (45.4%) had coronary artery disease. For both groups, the median (IQR) Morisky score was 5.5 (4.0-7.8), and the median (IQR) MLHFQ score was 43.0 (36.0-54.8) in the entire sample.

**Table 1.** Baseline Characteristics of Participants

Characteristic	Patients, No. (%)		
	All (N = 445)	Intervention group (n = 223)	Control group (n = 222)
Age, mean (SD), y	63.2 (13.3)	62.0 (13.30)	64.3 (13.1)
Sex			
Male	263 (59.1)	129 (57.8)	134 (60.4)
Female	182 (40.9)	94 (42.2)	88 (39.6)
BMI, mean (SD)	23.5 (6.6)	23.6 (6.9)	23.4 (6.3)
SBP, mean (SD), mm Hg	128.3 (23.9)	126.8 (24.4)	129.8 (23.3)
DBP, mean (SD), mm Hg	81.5 (33.6)	83.0 (44.9)	80.0 (15.8)
Heart rate, mean (SD), bpm	81.8 (19.8)	82.3 (21.5)	81.3 (18.0)
Smoking status			
No smoking	323 (72.6)	159 (71.3)	164 (73.9)
Current smoking	63 (14.2)	34 (15.2)	29 (13.1)
Quit smoking	57 (12.8)	28 (12.6)	29 (13.1)
Drinking status			
No drinking	349 (78.4)	174 (78)	175 (78.8)
Current drinking	40 (9)	21 (9.4)	19 (8.6)
Quit drinking	53 (11.9)	26 (11.7)	27 (12.2)
NYHA class^a^			
I	7 (1.6)	4 (1.8)	3 (1.4)
II	32 (7.2)	16 (7.2)	16 (7.2)
III	124 (27.9)	67 (30.0)	57 (25.7)
IV	209 (47.0)	99 (44.4)	110 (49.5)
LVEF, %			
<40	183 (41.1)	91 (40.8)	92 (41.4)
40-49	114 (25.6)	58 (26.0)	56 (25.2)
≥50	148 (33.3)	74 (33.2)	74 (33.3)
Medical history			
Valvular disease	42 (9.4)	22 (9.9)	20 (9.0)
Hypertension	246 (55.3)	124 (55.6)	122 (55.0)
Hyperlipidemia	22 (4.9)	10 (4.5)	12 (5.4)
CAD	202 (45.4)	97 (43.5)	105 (47.3)
Diabetes	97 (21.8)	53 (23.8)	44 (19.8)
Cardiomyopathy	61 (13.7)	32 (14.3)	29 (13.1)
AMI	17 (3.8)	7 (3.1)	10 (4.5)
Arrhythmia cordis	97 (21.8)	44 (19.7)	53 (23.9)
TIA	42 (9.4)	20 (9.0)	22 (9.9)
Medication			
ACEI or ARB	114 (25.6)	62 (27.8)	52 (23.4)
Diuretics	367 (82.5)	180 (80.7)	187 (84.2)
β-blockers	368 (82.7)	184 (82.5)	184 (82.9)
MRA	374 (84.0)	190 (85.2)	184 (82.9)
ARNI	245 (55.1)	127 (57.0)	118 (53.2)
SGLT-2i	109 (24.5)	53 (23.8)	56 (25.2)
Lipid-modifying agent	296 (66.5)	150 (67.3)	146 (65.8)
Cardiac glycoside	175 (39.3)	89 (39.9)	86 (38.7)
Antidepressant	66 (14.8)	32 (14.3)	34 (15.3)
Antithrombotic agent	378 (84.9)	190 (85.2)	188 (84.7)
Oral antidiabetic or insulin	167 (37.5)	89 (39.9)	78 (35.1)
Other medications	394 (88.5)	204 (91.5)	190 (85.6)
Laboratory measurements			
ALT, median (IQR), U/L	22.0 (14.0-36.4)	21.0 (13.9-40.8)	22.0 (14.0-33.2)
AST, median (IQR), U/L	24.0 (17.2-33.8)	24.0 (17.0-34.0)	24.0 (17.6-33.3)
Serum creatinine, median (IQR), mg/dL	0.9 (0.7-1.1)	0.9 (0.7-1.1)	0.9 (0.8-1.1)
BUN, median (IQR), mg/dL	18.2 (14.6-24.4)	18.2 (14.3-23.0)	18.2 (14.6-25.2)
LDL cholesterol, median (IQR), mg/dL	96.5 (73.4-119.7)	96.5 (77.2-119.7)	92.7 (73.4-119.7)
LVEDD, median (IQR), mm	54.0 (49.0-63.0)	54.0 (49.0-63.0)	54.0 (49.0-63.0)
LVESD, median (IQR), mm	39.0 (32.0-53.0)	40.0 (32.0-55.0)	38.5 (32.0-51.0)
LVEF, median (IQR), %	44.0 (33.3-54.8)	44.0 (33.8-52.3)	44.0 (33.0-55.0)
NT-proBNP, median (IQR), pg/mL	2563.5 (954.8-6558.1)	2516.5 (1068.2-6676.8)	2668.2 (714.5-6141.0)
BNP, median (IQR), pg/mL	379.7 (118.8-891.0)	416.0 (124.0-951.6)	374.4 (98.5-851.0)
Morisky scores, median (IQR)	5.5 (4.0-7.8)	5.5 (4.0-7.8)	5.5 (4.0-8.0)
MLHFQ scores, median (IQR)	43.0 (36.0-54.8)	42.0 (36.0-53.0)	44.0 (36.8-55.0)

Abbreviations: ACEI, angiotensin-converting enzyme inhibitor; ALT, alanine aminotransferase; AMI, acute myocardial infarction; ARB, angiotensin receptor blocker; ARNI, angiotensin receptor–neprilysin inhibition; AST, aspartate aminotransferase; BMI, body mass index (calculated as weight in kilograms divided by height in meters squared); BNP, B-type natriuretic peptide; bpm, beats per minute; BUN, serum urea nitrogen; CAD, coronary artery disease; DBP, diastolic blood pressure; LDL, low-density lipoprotein; LVEDD, left ventricular end-diastolic diameter; LVESD, left ventricular end-systolic diameter; LVEF, left ventricular ejection fraction; MLHFQ, Minnesota Living with Heart Failure Questionnaire (score range: 0-105, with the highest score indicating lowest quality of life); MRA, mineralocorticoid receptor antagonist; NYHA, New York Heart Association (class I or II [indicating mild limitations in physical activity] vs III or IV [indicating severe limitations in physical activity]); NT-proBNP, N-terminal pro–B-type natriuretic peptide; SBP, systolic blood pressure; SGLT-2i, sodium-glucose cotransport protein 2 inhibitors; TIA, transient ischemic attack.

SI conversion factors: To convert ALT and AST to microkatal per liter, multiply by 0.0167; BNP to nanogram per liter, multiply by 1.0; BUN to millimoles per liter, multiply by 0.357; LDL cholesterol to millimoles per liter, multiply by 0.0259; serum creatinine to micromoles per liter, multiply by 88.4.

^a^ Values may not sum due to missing data. Baseline data were extracted from the medical records; however, this classification field is not required to be completed by physicians in China.

In this study, the calculation of PDC followed the steps outlined in previous research.^16^ Compared with the control group, the intervention group showed significant improvement in PDC for heart failure treatment at 52 weeks (8.1%; 95% CI, 5.5%-10.7%; *P* < .001) (Table 2). The PDC for diuretics was significantly higher in the intervention group vs the control group (11.6%; 95% CI, 7.1%-16.1%; *P* < .001). The differences in PDC for β-blockers (4.6%; 95% CI, 0.2%-9.0%; *P* = .04) and MRAs (12.8%; 95% CI, 8.5%-17.2%; *P* < .001) were also significant; in contrast, there was no significant difference in PDC for ACEIs or ARBs. The proportion of patients with a PDC of 80% or greater among patients with CHF was significant (odds ratio, 2.94; 95% CI, 1.85-4.67; *P* < .001). However, there were no statistically significant differences in the proportions of patients achieving adherence (PDC ≥80%) for ACEIs or ARBs and β-blockers. After application of the Bonferroni correction, although the calculated *P* values for diuretics and MRAs showed significant differences when compared with the corrected *P* value (.01), the conclusions for all secondary end points were consistent with the preliminary analysis results, indicating the robustness of the results.

**Table 2.** Primary and Secondary Outcomes

Outcome	Intervention group		Control group		Intervention effect (95% CI), %	OR (95% CI)	NNT	*P* value
	No. (%)	Mean (SD), %	No. (%)	Mean (SD), %				
**Primary **								
Mean PDC	223 (50.1)	92.9 (11.3)	222 (49.9)	84.8 (16.4)	8.1 (5.5 to 10.7)	NA	NA	<.001
**Secondary **								
Mean PDC ≥80%	190 (85.2)	NA	147 (66.2)	NA	NA	2.94 (1.85-4.67)	5.27	<.001
ACEIs or ARBs								
PDC	62 (54.4)^a^	86.5 (28.8)	52 (45.6)^a^	88.6 (27.5)	−2.2 (−12.7 to 8.4)	NA	NA	.69
PDC ≥80%	49 (79.0)^b^	NA	44 (84.6)^b^	NA	NA	0.69 (0.26-1.81)	17.9	.44
Diuretics								
PDC	180 (49.0)	94.6 (15.6)	187 (51.0)	83.0 (26.5)	11.6 (7.1 to 16.1)	NA	NA	<.001
PDC ≥80%	161 (89.4)	NA	126 (67.4)	NA	NA	4.10 (2.33-7.22)	4.5	<.001
β-blockers								
PDC	184 (50.0)	95.2 (17.9)	184 (50.0)	90.5 (24.5)	4.63 (0.23 to 9.04)	NA	NA	.04
PDC ≥80%	170 (92.4)	NA	159 (86.4)	NA	NA	1.91 (0.96-3.80)	16.7	.06
MRAs								
PDC	190 (50.8)	96.6 (12.1)	184 (49.2)	83.8 (27.9)	12.8 (8.5 to 17.2)	NA	NA	<.001
PDC ≥80%	178 (93.7)	NA	134 (72.8)	NA	NA	5.54 (2.84-10.8)	4.8	<.001

Abbreviations: ACEI, angiotensin-converting enzyme inhibitor; ARB, angiotensin receptor blocker; MRA, mineralocorticoid receptor antagonist; NA, not applicable; NNT, number needed to treat; OR, odds ratio; PDC, proportion of days covered.

^a^ The number of patients for each heart failure medication's PDC, and the proportion of patients in the control/intervention group relative to the total group for each heart failure medication.

^b^ The total number of patients receiving ACEIs or ARBs, and the proportion of patients who have PDC ≥ 80% relative to the total number of patients receiving ACEIs or ARBs.

Compared with the control group, the quality of life at 52 weeks was significantly improved in the intervention group, as reflected by a statistically meaningful reduction in median (IQR) MLHFQ scores (25.0 [12.0-40.0] vs 35.0 [20.0-44.0] points; difference, 10.0 points; *P* = .005) (eTable 1 in Supplement 2). We found a statistically significant difference in median (IQR) Morisky scores between the groups (7.8 [6.8-8.0] vs 7.3 [4.5-7.8] points; difference, 0.5 points; *P* < .001) (eTable 1 in Supplement 2), although no significant difference in safety outcomes was observed (eTable 2 in Supplement 2). Among the 445 participants, 165 (37.1%) experienced ADEs, of which 131 were judged as unrelated or unlikely to be related to the study. Among the 34 possibly or definitely related ADEs, 15 occurred in the control group (6 cases of gastrointestinal reactions, 4 male gynecomastia, 2 shortness of breath, 2 bleeding, and 1 liver injury).

**Figure 2.**
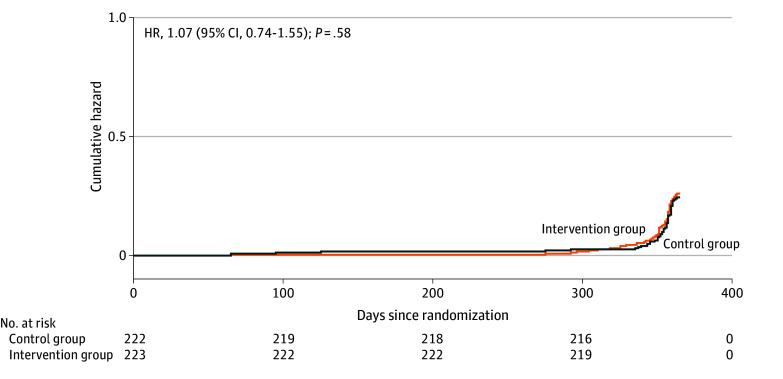
Nelson-Aalen Plot for Time to First Event During 52-Week Follow-Up

Within 52 weeks after randomization, 4 patients in the intervention group and 7 patients in the control group died. No significant differences between the 2 groups were observed in readmission rates, which were defined as unplanned cardiovascular hospitalizations, or all-cause deaths. However, there was no significant difference in the number of days lost due to unplanned cardiovascular hospitalizations or deaths between the 2 groups (eTable 3 in Supplement 2). The Nelson-Aalen plot for all-cause mortality or unplanned cardiovascular hospitalizations within 52 weeks after randomization is shown in Figure 2.

**Figure 3.**
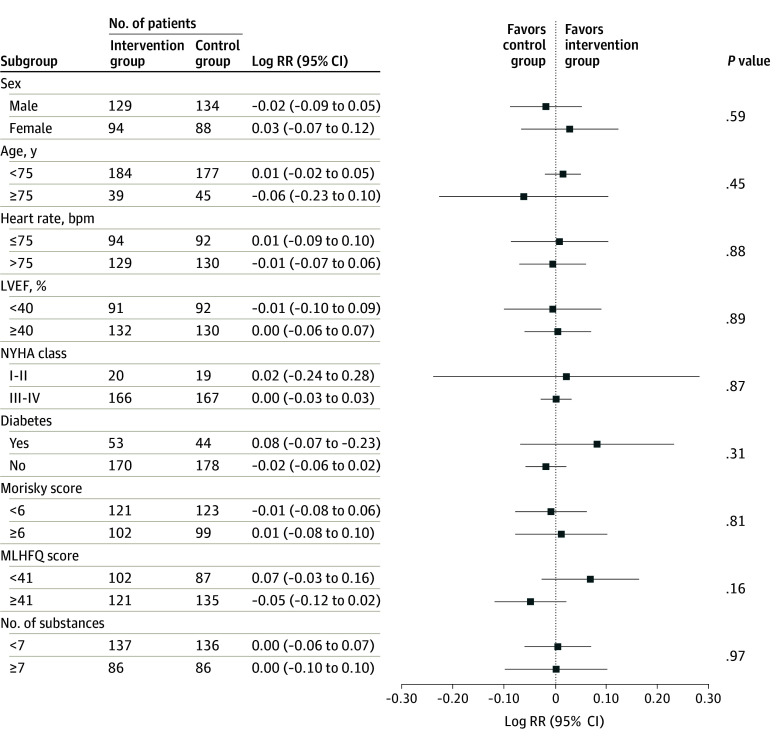
Forest Plot of Sensitivity Analyses for the Primary Efficacy End Point bpm indicates beats per minute; LVEF, left ventricular ejection fraction; MLHFQ, Minnesota Living With Heart Failure Questionnaire; NYHA, New York Heart Association; and RR, relative risk.

Among all subgroups examined, we identified 9 factors that had the potential to influence the primary efficacy end point. As previously mentioned, the intervention group compared with the control group showed improved medication adherence at 52 weeks. However, no significant interactions were observed across all subgroups (Figure 3).

## Discussion

To our knowledge, this randomized clinical trial in the Chinese population was the first to investigate the effect of a multifaceted and interdisciplinary intervention led by clinical pharmacists on the management of patients with CHF. Although several trials have assessed the effectiveness of pharmacists in the treatment of patients with CHF, these trials have not been methodologically consistent.^16,28,29,30,31,32^ Their key conclusions suggest that the role of pharmacists in improving patient adherence and clinical outcomes is limited. Our findings are largely consistent with those of the past trials. While there was a statistically significant improvement in medication adherence among patients with CHF under the pharmacist-led management model, the improvement was modest. Moreover, no significant differences were observed in clinical outcomes such as readmission rates, all-cause deaths, and ADEs. We believe this finding may be due to the limited clinical effectiveness of the pharmacist intervention or the relatively short observation period, neither of which was sufficient to result in significantly different clinical outcomes. This finding suggests that more effective interventions need to be explored in future research to achieve substantial improvements in patient health outcomes. Considering that the trial’s recruitment period (March 2021 to April 2022) overlapped with the peak of the COVID-19 outbreak in Hebei Province by 2 months, we noticed a reduction in the number of new patients reported by the participating hospitals during that period. Additionally, the consultation time during follow-up visits was extended, and the frequency of medication changes increased. However, we observed that possibly due to the collective anxiety triggered by the COVID-19 pandemic, medication adherence in both groups of participants improved, a finding consistent with reports from health care workers in France and Switzerland.^33,34^ We speculate that this phenomenon of increased adherence under stress may be one of the factors in the high medication adherence observed in both the intervention and control groups in this trial.

Heart failure imposes considerable limitations on quality of life, and the success of heart failure treatment is closely related to improvements in quality of life.^35^ Poor quality of life among patients with heart failure is associated with an increased risk of all-cause mortality and a higher combined risk of heart failure–related death or hospitalization.^36^ Rasmussen et al^37^ found that patients with CHF who are in the terminal stage of various heart diseases often have comorbidities, and medication adherence is closely related to quality of life. This finding underscores the necessity of research such as the current trial. During the COVID-19 pandemic, the MLHFQ scores in the intervention group at 52 weeks were significantly reduced compared with baseline and significantly lower than MLHFQ scores in the control group. This result could potentially elucidate the benefit of messaging application–based follow-up frequency for the long-term outcomes of patients with CHF.

Patients with CHF are often older and have multiple comorbidities. Thus, they typically require the use of combined multiple medications, which increases the risk of drug-related adverse events.^38,39^ Based on their professional pharmaceutical knowledge, clinical pharmacists can provide answers to medication inquiries and offer guidance on dealing with adverse reactions, thereby reducing the occurrence of ADEs. In this study, there was no significant difference in the incidence of ADEs between the intervention and control groups. Similarly, Gurwitz et al^40^ found that clinical pharmacist-led interventions did not significantly reduce the incidence of drug-related adverse events or clinically significant medication errors. Other studies have indicated that pharmacist-led interventions may reduce the incidence of adverse reactions in chronic diseases.^41,42,43^ However, our findings underscore the necessity for larger sample sizes to confirm whether such interventions can decrease the prevalence of adverse reactions among patients with CHF.

The messaging application used in this trial is widely used in China, suggesting that the application may be useful for implementing mobile health interventions to improve medication adherence. Although some studies have been conducted in China using this application to improve self-management behaviors,^22,24,44,45^ none of them has specifically addressed medication adherence among patients with CHF. In this study, we leveraged the messaging application, a familiar platform for patients, to implement clinical pharmacist interventions in which pharmacists communicated with patients with CHF at any time. Educational materials on medication were regularly posted in the application’s chat groups, and weekly medication reminders prompted patients to take their medication. These measures were particularly important for patients who were confined to their homes during the COVID-19 pandemic. We speculate that the beneficial effects of the pharmacist-led management model may be related to the messaging application–based education. Previous studies have shown that the messaging application’s platform can provide individualized guidance, thereby enhancing patient adherence,^26,46^which is consistent with our findings. The scope of this trial can be extended to other terminal illnesses to alleviate the economic and lifestyle burdens of these serious diseases on patients and society. Furthermore, our experience with this pharmacist-led intervention has provided a standardized pathway for training grassroots pharmacists in rural areas of China, especially in the field of heart failure management, which helps to improve the quality of treatment and patient care levels in these regions.

### Limitations

This study has several limitations. First, all participants were recruited from 5 hospitals in Hebei province. Although hospitals of different levels were included, the sample still may not be representative of the general population in China. Second, due to the uniqueness of the COVID-19 pandemic, some participants self-reported their medication use, which may have introduced variability in the results. Third, during data collection for the control group, some participants requested to communicate with nurses through the messaging application. However, we did not consider this effect in our analysis. Fourth, the absence of an attention control group may affect the accuracy of the assessment of the impact of the pharmacist-led management model on patients with heart failure.

## Conclusions

In this randomized clinical trial, patients with CHF who received the pharmacist-led management intervention showed a significant improvement in medication adherence compared with patients who received usual care, but the improvement was modest. No significant differences were observed between these groups in terms of clinical outcomes, including readmission rates, all-cause mortality, and ADEs. Future research may need to examine more comprehensive intervention strategies to identify the impact of pharmacists on the incidence and mortality of CHF.

## Notes

Supplement 1.
Trial Protocol and Statistical Analysis Plan

Supplement 2. **eTable 1.** The Outcomes of Morisky Scores and MLHFQ Scores **eTable 2.** Safety Outcomes **eTable 3.** All-Cause Deaths and Unplanned Cardiovascular Hospitalizations During 52-Week Follow-Up

Supplement 3.
Data Sharing Statement
