# Errors in the Abstract, Results, and Table 2

**DOI:** 10.1001/jamanetworkopen.2025.11780

**Published:** 2025-04-18

In the Original Investigation titled, “Pharmacist-Led Management Model and Medication Adherence Among Patients With Chronic Heart Failure: A Randomized Clinical Trial,”^1^ published online December 20, 2024, there were errors in the odds ratios reported in the Abstract, Results, and Table 2. In the Abstract, the final sentence of the Results should be the following: “At 52 weeks, the intervention group had a significantly higher PDC for heart failure medication (8.1%; 95% CI, 5.5%-10.7%; *P* < .001) and a greater proportion of patients with PDC of 80% or greater (odds ratio, 2.94; 95% CI, 1.85-4.67; *P* < .001) compared with the control group.” In the second paragraph of the Results section in the text, the fifth sentence should be the following: “The proportion of patients with a PDC of 80% or greater among patients with CHF was significant (odds ratio, 2.94; 95% CI, 1.85-4.67; *P* < .001).” In Table 2, the odds ratios were incorrect. This article has been corrected.
