# Errors in Table 2

**DOI:** 10.1001/jamanetworkopen.2026.13456

**Published:** 2026-04-22

In the Original Investigation titled, “Pharmacist-Led Management Model and Medication Adherence Among Patients With Chronic Heart Failure: A Randomized Clinical Trial,”^1^ published online December 20, 2024, the mean (SD) percentage was incorrect for the intervention and control groups in the mean proportion of days covered (PDC) row of Table 2. In the mean PDC row, the correct mean (SD) percentage for the intervention group should be 92.9 (11.3) and 84.8 (16.4) for the control group. This article has been corrected.
