# Supplementary material for: Pharmacist-Led Management Model and Medication Adherence Among Patients With Chronic Heart Failure: A Randomized Clinical Trial
Source: JAMA Netw Open. 2024 Dec 20;7(12):e2453976. doi: 10.1001/jamanetworkopen.2024.53976 (PMC11662253; doi:10.1001/jamanetworkopen.2024.53976)
Supplement: Supplement 1. — Trial Protocol and Statistical Analysis Plan [file jamanetwopen-e2453976-s001.pdf]

The protocol version to 2.0

The protocol version to 1.0

Statistical Analysis Plan 1.0

Pharmacist-led management versus conventional physician/nurse management in medication adherence and efficacy among patients with chronic heart failure: A multicenter, randomized controlled study

Applicant Institution: The First Hospital of Hebei Medical University

Principal Investigator:Lingjiao Wang

Version 2.0

August 4, 2021

Researcher Consent Form

I agree to:

Take responsibility for the correct implementation of clinical research at our center.

Follow the research protocol, protocol amendments, and ethical committee requirements for conducting clinical trials.

Conduct the clinical study in compliance with Good Clinical Practice (GCP) guidelines and regulatory requirements.

Ensure that all personnel at our center involved in this study are fully acquainted with the study procedures, methods, investigational drugs involved, and their respective roles in the study.

Not modify or implement the research protocol without approval from the ethics committee, except when necessary to take urgent actions to minimize harm to subjects or for research management purposes (subject to approval by relevant regulatory authorities).

Researcher Signature / Date

____

Researcher Name (in block letters)

____

Research Institution

____

Contact Phone Number

____

Protocol Modifications (more recent changes listed first)

Date	Summary of Changes
04/08/2021	● Changed the protocol version to 2.0.<br>● Added the following evaluation indicators: The Morisky Medication Adherence Scale, days lost due to unplanned cardiovascular hospitalizations and death.<br>● Removed the following evaluation indicators: NT-proBNP/BNP levels; differences in cardiac function indicators (e.g., LVDd, LVSD, LVEF) from baseline; NYHA functional classification at week 52; frequency of adverse drug events.<br>● Added subgroup analysis.
08/07/2021	● Add Cangzhou Central Hospital as a study site (Obtained approval from the Ethics Committee ofCangzhou Central Hospital).<br>● Removal of Tangshan Workers' Hospital and Tangshan Kailuan General Hospital due to unclear ethical approval.
20/04/2021	● Add the First Hospital of Handan as a study site (Obtained approval from the Ethics Committee of the First Hospital of Handan)<br>● Due to varying COVID-19 situations in each center's city, the addition of telemedicine services for doctors is implemented.
03/03/2021	● Add the Fourth Hospital of Handan as a study site (Obtained approval from the Ethics Committee of the Fourth Hospital of Handan)

Summary of Protocol

Title	Pharmacist-led management versus conventional physician/nurse management in medication adherence and efficacy among patients with chronic heart failure:<br>A multicenter, randomized controlled study
Objective	1. Compare the difference in medication adherence between pharmacist-led management and conventional physician/nurse management in patients with chronic heart failure.<br>2. Evaluate the effectiveness and safety of pharmacist-led management versus conventional physician/nurse management in the treatment of chronic heart failure.
Study Design	Multicenter, Randomized Controlled Study
Principal Investigator	Lingjiao Wang(The First Hospital of Hebei Medical University); Baogui Chong (Hengshui People's Hospital); Liping Han (Handan First Hospital); Huan Zhang (Cangzhou Central Hospital); Aixia Liu (The Fourth Hospital of Handan).
Number of Centers	5
Estimated Number of Patients to be Included	Based on previous studies (Schulz, M., et al. European Journal of Heart Failure, 2019. 21(8): p. 1012-1021), the overall standard deviation of PDC for heart failure medication at 52 weeks is 14.9. Assuming a mean difference of 4.0 between the two groups, with α set at 0.05 and β at 0.20, each group would require 218 participants, accounting for an expected attrition rate of 10%. Thus, each group would need 243 participants, totaling 486 participants overall.
Duration of the Study	3 years
Patient Recruitment Period	November 2020 to November 2023 (subject to adjustment based on case accrual).
Study Population	Inclusion Criteria (all must be met):<br>1.Age ≥ 18 years old;<br>2.Diagnosed with chronic heart failure;<br>3.Stable on an established pharmacological treatment regimen for heart failure;<br>4.Subjects able to understand and sign the informed consent form.
	Exclusion Criteria (any of the following):<br>1.Inability to comply with study-related assessments/follow-ups;<br>2.Planned cardiac surgery during the study period;<br>3.Life expectancy less than 1 year;<br>4.History of malignant neoplasms;<br>5.Severe hepatic or renal dysfunction (liver enzymes > 3 times the upper limit of normal or estimated glomerular filtration rate < 30 mL/min);<br>6.Participation in another clinical study within the past three months or currently participating;<br>7.Pregnant or lactating women.
	Withdrawal Criteria (any of the following):

	1.Occurrence of adverse events (including serious adverse events) deemed by the investigator as unsuitable to continue participation in the clinical study;<br>2.Any situation where the investigator considers poor compliance;<br>3.Loss to follow-up;<br>4.Subject withdraws informed consent.
	Discontinuation Criteria (any of the following):<br>1.Diagnostic error where the subject does not meet the indications for this study. The subject is excluded from the efficacy dataset but all safety observations are retained in the safety dataset.<br>2.Subjects who have been enrolled in the study but have not received any interventions.
Baseline Indicators	Baseline indicators include the following:<br>1. Demographic data (such as age, sex, race, etc.);<br>2. Medical history (including past illnesses, surgical history, etc.);<br>3. Smoking and alcohol history;<br>4.Vital signs (such as blood pressure, heart rate, etc.);<br>5. Diagnosis time and etiology of heart failure;<br>6. Current medications;<br>7.Minnesota Living with Heart Failure Questionnaire (MLHFQ) score;<br>8. Morisky Medication Adherence Scale score;<br>9.New York Heart Association (NYHA) functional classification;<br>10. Complete blood count (CBC);<br>11. Liver function tests;<br>12. Kidney function tests;<br>13. Electrolyte levels;<br>14. Fasting blood glucose;<br>15. Glycated hemoglobin (HbA1c);<br>16.Lipid profile (including total cholesterol, triglycerides, LDL-C, HDL-C, etc.)<br>17. N-terminal pro-B-type natriuretic peptide (NT-proBNP) or B-type natriuretic peptide (BNP) levels;<br>18. Chest X-ray findings;<br>19.Echocardiogram results (including left ventricular end-diastolic diameter (LVDd), left ventricular end-systolic diameter (LVDs), and left ventricular ejection fraction (LVEF)).<br>These baseline indicators are used to assess the health status and disease characteristics of patients prior to entry into clinical studies.
Grouping, intervention, and blinding:	Based on LVEF stratification: heart failure with reduced ejection fraction (LVEF $<$ 40%), heart failure with mid-range ejection fraction (LVEF $\geqslant$ 40% and $<$ 50%), and heart failure with preserved ejection fraction (LVEF $\geqslant$ 50%), totaling 3 strata. Using a computer-generated random number table, subjects are randomized 1:1 into a pharmacist-led group and a

	traditional care group using opaque envelopes for concealed allocation. Blinding is not implemented for researchers and patients in this study, but the assessors are blinded. All interventions commence within 24 hours prior to discharge.
Follow-up	In this study, patient follow-up will be conducted by trained and designated study personnel. Blinding of assessors will be implemented, where the assessors will not know the specific group assignments of the patients they are evaluating.<br>Patients will be followed up every 4 weeks for a total of 52 weeks in this study. The follow-up will involve recording the number of missed days for heart failure treatment medications (beta-blockers, ACEI/ARB, mineralocorticoid receptor antagonists, diuretics, etc.). Every 4 weeks, blood tests including complete blood count, liver and kidney function tests, electrolytes, lipid profile, NT-proBNP/BNP levels, and echocardiography will be conducted.<br>At the final follow-up, assessments will include NYHA functional classification, Morisky Medication Adherence Scale, and Minnesota Living with Heart Failure Questionnaire (MLHFQ). The overall and individual medication class-specific Proportion of Days Covered (PDC) will be calculated. Adverse events, non-cardiovascular rehospitalizations, and all-cause mortality events will be recorded, with the researcher determining whether adverse events are treatment-related.
Outcome	Primary efficacy endpoint: Overall Proportion of Days Covered (PDC) with heart failure medications at 52 weeks.<br>Secondary efficacy endpoints: Individual PDC for each class of treatment-related medications at 52 weeks; proportion of patients with overall and class-specific PDC ⩾ 80%; MLHFQ score at week 52; Morisky Medication Adherence Scale score; non-cardiovascular rehospitalization; all-cause mortality composite endpoint events, and days lost due to unplanned CV hospitalizations and death.<br>Safety endpoints: Number of adverse drug events. Common adverse events include hyperglycemia, electrolyte disturbances, sleep disorders, mood disorders, atrial fibrillation, ventricular tachycardia, acute coronary syndrome, infections, worsening renal function, dialysis, cardiogenic shock, and syncope.
Statistical analysis methods	Statistical analysis was performed using SPSS 20.0 software.<br>2. The study utilized the full analysis set (patients who received at least one treatment and underwent at least one efficacy assessment) for efficacy analysis, and the safety set (patients who received at least one treatment and underwent at least one safety assessment) for safety analysis. Missing data were handled using the last observation carried forward (LOCF) method.<br>3. Continuous variables were described as mean ± standard deviation if normally distributed, or as median (interquartile range) if non-normally distributed. Categorical variables were presented as counts (percentages).

	4. Between-group comparisons of continuous variables were conducted using independent samples t-test or Mann-Whitney U test. Categorical variables were compared using chi-square test or exact probability method.<br>5. Nelson-Aale survival curves were plotted for the composite endpoint of heart failure hospitalization and death. Group differences were assessed using the log-rank test.<br>6. Subgroup analyses will be conducted for the primary and secondary efficacy endpoints to assess the consistency of intervention effects across the following subgroups: age (<75 vs. ≥75 years), gender, heart rate at baseline (≤75 vs. >75 b.p.m.), NYHA class (I/II vs. III/IV), left ventricular ejection fraction (LVEF, <40% vs. ≥40%), history of diabetes, level of disease burden (number of different medications at baseline), baseline adherence, and quality of life at baseline.<br>7. statistical significance set at $P < 0.05$.

1 Background

Chronic heart failure (CHF) is a complex clinical syndrome characterized by chronic structural and functional abnormalities of the heart. It represents a common outcome of acute and chronic cardiac

conditions. Specifically, CHF refers to a persistent state of heart failure, typically presenting with symptoms such as dyspnea, fatigue, and fluid retention. The condition can exhibit a stable course, worsen over time, or become decompensated[1, 2]. According to reports, the prevalence of chronic heart failure (CHF) among adults in China ranges from 0.9% to 4.6%. As age increases, the incidence of CHF rises rapidly [3]. The main causes of heart failure in China include coronary heart disease, hypertension, dilated cardiomyopathy, valvular heart disease, and diabetes, among others [2-6]. Previous studies have shown that with the advancement of medical care in China, the in-hospital mortality rate of heart failure patients has significantly decreased [4, 6-8]. For instance, recent research indicates that the in-hospital mortality rate for hospitalized heart failure patients is approximately 4.1% [4], with a one-year mortality rate around 7.8%. However, the rate of heart failure readmissions is reported to be as high as 30% [6]. Therefore, as China's population ages and the prevalence of heart failure risk factors such as coronary heart disease, hypertension, and diabetes increases, coupled with improved medical care prolonging the survival of cardiac patients, the incidence of CHF is expected to continue rising [2, 3]. This trend will impose a significant burden on individuals and the healthcare system alike. Hence, urgent efforts are needed in the prevention and treatment of CHF.

Currently, drug therapy is the preferred method for treating CHF [1, 2, 9]. However, recent studies indicate that approximately 40% to 80% of CHF patients exhibit poor medication adherence, including under-dosing, missing doses, or discontinuing medications [10, 11]. Poor medication adherence is a significant factor leading to adverse outcomes in CHF patients, such as hospital readmissions [12]. In addition, besides the medications recommended by guidelines, CHF patients often require additional drug treatments due to comorbidities and complications. This results in polypharmacy, increasing the risks of drug-drug interactions, adverse drug reactions, and poor adherence to medication [13-15].

Therefore, it is necessary to take measures to improve patient medication adherence and rationalize drug therapy to enhance treatment outcomes.

Several pharmacist-led or pharmacotherapy-based interventions for CHF patients conducted abroad [16-19] have shown that clinical pharmacists intervening in clinical settings can improve medication adherence, as well as enhance drug efficacy and safety. In China, a few studies have explored the impact of clinical pharmacist interventions on improving medication adherence, treatment outcomes, and quality of life in chronic heart failure patients, yielding conclusions similar to those observed in the aforementioned foreign studies [20-25]. In a randomized controlled trial [25], 100 chronic heart failure patients at risk of medication non-adherence were enrolled and randomly divided into two groups, each consisting of 50 patients. The control group received routine bedside and discharge medication guidance from doctors and nurses. In contrast, the intervention group received additional medication adherence interventions and guidance from clinical pharmacists at the hospital, in addition to routine medication instructions. The study compared medication adherence, clinical appropriateness of drug use, readmission rates, and mortality between the two groups. The results indicated that at 1 month and 3 months post-discharge, medication adherence in the intervention group was better than that in the control group (74% vs 52%; 94% vs 60.0%, respectively). Additionally, beyond 3 months post-discharge, the intervention group showed lower rates of clinically inappropriate drug use and readmission compared to the control group (14 cases vs 24 cases; 14% vs 32%). The previous domestic studies have the following limitations: mostly small sample sizes (less than 200 cases) and single-center studies; greater focus on short-term adherence and safety efficacy indicators; clinical pharmacists often participate in related studies as participants rather than leaders. Therefore, it is necessary to conduct a multicenter, randomized controlled study with a large sample size to compare high-quality pharmacist-led management models

with routine physician/nurse management models regarding long-term medication adherence and efficacy differences in patients with chronic heart failure.

It has been reported that WeChat has become China's most popular instant messaging application, with a monthly active user base reaching as high as 549 million [26]. WeChat's widespread adoption, timeliness, and convenience make it highly suitable for use in public health interventions in China. Numerous studies have already utilized WeChat for patient disease management, such as in diabetes [26], HIV/AIDS [27], demonstrating positive outcomes. Emerging social media platforms offer promising new methods for managing chronic health conditions. Recently, several studies have explored the continuous follow-up and guidance of heart failure patients using the WeChat platform [29-31]. The results indicate that these emerging WeChat follow-up methods help improve medication adherence among heart failure patients, promote clinically appropriate medication use, and enhance patient outcomes. In addition, establishing WeChat groups to disseminate relevant health education content about heart failure has been shown to guide discharged heart failure patients in home-based rehabilitation. This approach helps improve compliance with home-based rehabilitation and enhances patients' self-care abilities, thereby potentially improving patient outcomes [30]. However, there is currently a lack of research investigating the impact of pharmacist-led management models using WeChat to remind patients about medication adherence and provide patient education on the compliance and effectiveness outcomes for CHF (Congestive Heart Failure) patients. Additionally, existing studies in China on WeChat's involvement in disease management for heart failure patients often rely on single-center studies with small sample sizes. Therefore, further validation through multicenter, large-sample, high-quality evidence is needed to substantiate these findings.

Based on the above, this study plans to conduct a multicenter, large-sample, open-label, pragmatic

randomized controlled trial. Patients with chronic heart failure will be enrolled, and their baseline data will be collected. Stratification will be done based on preserved ejection fraction heart failure (HFpEF), reduced ejection fraction heart failure (HFrEF), and mid-range ejection fraction heart failure (HFmrEF). Participants will be randomly assigned to either a pharmacist-led intervention group or a conventional physician/nurse management group. Follow-up will be conducted for 52 weeks to compare the pharmacist-led intervention group with the conventional management group. Key outcomes will include medication possession ratio (PDC), incidence of mortality, and unplanned cardiovascular admissions. These measures aim to assess the impact of pharmacist-led intervention on medication adherence (non-pharmacological factors) and therapeutic efficacy (pharmacological factors) in chronic heart failure.

2 Study aims

(1) Comparison of differences in medication adherence among chronic heart failure patients under pharmacist-led management versus conventional physician/nurse management.

(2) Comparison of the effectiveness and safety of chronic heart failure treatment under pharmacist-led management versus conventional physician/nurse management.

3 Study participants

3.1 Inclusion criteria

All of the following entries are met:

(1) Age ≥ 18 years old;

(2) Diagnosed with chronic heart failure;

(3) Stable on an established pharmacological treatment regimen for heart failure;

(4) Subjects able to understand and sign the informed consent form.

3.2 Exclusion criteria

Exclclusion to any entry:

(1) Inability to comply with study-related assessments/follow-ups;

(2) Planned cardiac surgery during the study period;

(3) Life expectancy less than 1 year;

(4) History of malignant neoplasms;

(5) Severe hepatic or renal dysfunction (liver enzymes > 3 times the upper limit of normal or estimated glomerular filtration rate < 30 mL/min);

(6) Participation in another clinical study within the past three months or currently participating;

(7) Pregnant or lactating women.

3.3 Withdrawal Criteria

Exit upon meeting any entry:

(1) Occurrence of adverse events (including serious adverse events) deemed by the investigator as unsuitable to continue participation in the clinical study;

(2) Any situation where the investigator considers poor compliance;

(3) Loss to follow-up;

(4) Subject withdraws informed consent.

3.4 Discontinuation Criteria

Any entry is exclusion:

a. Diagnostic error where the subject does not meet the indications for this study. The subject is excluded from the efficacy dataset but all safety observations are retained in the safety dataset.

b. Subjects who have been enrolled in the study but have not received any interventions.

Based on the above inclusion and exclusion criteria, patients were continuously recruited from the

cardiology departments of the five hospitals: the First Hospital of Hebei Medical University, Hengshui People's Hospital, the First Hospital of Handan, Cangzhou Central Hospital, and the Fourth Hospital of Handan.

The study was conducted in accordance with the Helsinki Declaration [33]. The study was approved by the ethics committees of the five hospitals mentioned above, and all participants signed informed consent forms.

4 Study design and treatment

4.1 Study design

The current project aims to conduct a large-sample, multicenter, open-label, superiority randomized controlled trial comparing the long-term medication adherence and treatment efficacy of pharmacist-led management versus conventional physician/nurse management for chronic heart failure patients. Patients with chronic heart failure will be enrolled, and their baseline data will be collected. They will be stratified based on heart failure with preserved ejection fraction (HFpEF), heart failure with reduced ejection fraction (HFrEF), and heart failure with mid-range ejection fraction (HFmrEF). Participants will be randomly divided into a pharmacist-led group and a traditional care group. The traditional care group will be managed by regular physicians and nurses, while the pharmacist-led group will receive pharmacist-directed interventions, utilizing WeChat-based patient management. After 52 weeks of follow-up, the medication possession ratio (PDC), mortality events, and unplanned cardiovascular hospitalizations will be compared between the two groups to evaluate the impact of pharmacist-led interventions on medication adherence (non-pharmacological factors) and treatment efficacy (pharmacological factors) for chronic heart failure patients.

4.2 Randomization, grouping and blindness

Stratification based on LVEF: Patients will be stratified into three groups: heart failure with reduced ejection fraction (LVEF < 40%), heart failure with mid-range ejection fraction (LVEF ≥ 40% and < 50%), and heart failure with preserved ejection fraction (LVEF ≥ 50%). Using a computer-generated random number table, participants will be randomly assigned in a 1:1 ratio to the pharmacist-led group or the traditional care group, with stratification based on the LVEF levels. Opaque envelopes will be used for concealed allocation.

This study will be open-label for both researchers and patients, but blinded for the assessors.

4.3 Clinical Intervention Program

All interventions were initiated within 24h before hospital discharge.

Traditional mode group

a. Baseline:

The attending physician will order tests and examinations based on the patient's clinical presentation. Based on the results, adjustments will be made to the medication regimen, including medications for heart failure, hypertension, lipid regulation, and diabetes. A prescription for a 4-week/1-month supply of medication will be issued, and patients will be instructed to take their medication regularly.

The nurse will print out a medication list, distribute heart failure educational brochures, and provide a medication diary. Patients will be informed to record each medication taken in the diary. An appointment for the next follow-up visit (every 4 weeks/1 month) will be scheduled.

b.Subsequent Follow-up Visits:

The nurse will remind and confirm the appointment time seven days before each visit.

The nurse will check the patient's medication diary and compare it with the actual prescription.

The attending physician will order tests and examinations based on the patient's clinical presentation.

Based on the results, adjustments will be made to the medication regimen, and a prescription for a 4-week supply of medication will be issued. Patients will be instructed to take their medication regularly.

The nurse will print out a medication list and remind the patient to record each medication taken in the diary. An appointment for the next follow-up visit will be scheduled.

During the follow-up process, if a patient is hospitalized, the physician will determine whether the hospitalization is related to heart failure or other adverse events during the subsequent visit. The follow-up visit will then continue according to the aforementioned process.

Pharmacist-Led group

a. Baseline:

Here is the translation of the Chinese text you sent into English:

The pharmacist will inspect all medication packages to understand the patient's actual adherence to the medication regimen and compare it with the prescription.

The attending physician will order tests and examinations based on the patient's clinical presentation. The physician will then discuss with the pharmacist regarding any necessary adjustments to the medication regimen, including medications for heart failure, hypertension, lipid regulation, and diabetes.

Based on the discussion, the physician will issue a prescription for a 4-week/1-month supply of medication.

The pharmacist will conduct patient education, explaining the treatment goals for each condition, the role and necessity of the medications, dosage and administration instructions, possible adverse events, management strategies, and drug-drug interactions.

The pharmacist will use medication boxes to dispense the medications, label them with dosage and administration instructions, print out the medication list, distribute heart failure educational brochures,

and provide a medication diary. Patients will be instructed to record each medication taken in the diary. An appointment for the next follow-up visit (every 4 weeks/1 month) will be scheduled.

The pharmacist will add the patient or their caregiver as a WeChat friend and create a patient group chat. The pharmacist will privately remind the patient once a week to take their medication and record it in the medication diary. If the patient or their caregiver needs medication or disease-related advice, they can privately message the pharmacist or use the group chat, and the pharmacist will provide a response.

b.Subsequent Follow-up Visits:

Seven days before the visit, the pharmacist will remind and confirm the appointment time.

During the visit, the pharmacist will check the patient's medication diary, compare it with the actual prescription, and inquire about any adverse events that occurred or any new medications taken.

The attending physician will order tests and examinations based on the patient's clinical presentation and discuss with the pharmacist any necessary adjustments to the medication regimen.

The physician will issue a prescription for a 4-week/1-month supply of medication.

The pharmacist will conduct patient education based on the medication adherence in the past 4 weeks/1 month, focusing on any incorrectly taken, missed, newly added, or adjusted medications. This education will cover the role, necessity, dosage and administration instructions, possible adverse events, management strategies, and drug-drug interactions of the medications.

The pharmacist will use medication boxes to dispense the medications, label them with dosage and administration instructions, and instruct the patient to record each medication taken in the diary. An appointment for the next follow-up visit will be scheduled.

During the follow-up process, if a patient is hospitalized, the physician will determine whether the hospitalization is related to heart failure or other adverse events during the subsequent visit. The follow-

up visit will then continue according to the aforementioned process.

5 Study planning and data collection

The original data to be collected for this study originates from the original medical records filled in by the researchers and the original records filled in by the research subjects. The researchers are required to guide the research subjects in accordance with the study protocol for the prescribed treatment, conduct follow-up visits at the scheduled times, and promptly organize and save all relevant records or results. The research subjects must fill in the corresponding data in a timely and detailed manner as requested by the researchers and promptly submit them for preservation.

5.1 Laboratory examination

Blood routine test, liver function, kidney function, electrolytes, glycosylated hemoglobin (HbA1c), blood lipids, N-terminal pro-B-type natriuretic peptide (NT-proBNP)/B-type natriuretic peptide (BNP), etc.

5.2 Baseline data and indicators

Demographic data, past medical history, smoking and alcohol history, vital signs, time of heart failure diagnosis, etiology, current treatment medications, Minnesota Living with Heart Failure Questionnaire (MLHFQ) score, Morisky Medication Adherence Scale, New York Heart Association (NYHA) functional class, blood routine test, liver and kidney function, electrolytes, fasting blood glucose, glycosylated hemoglobin (HbA1c), blood lipids, NT-proBNP/BNP, chest X-ray, echocardiography including left ventricular end-diastolic diameter (LVDd), left ventricular end-systolic diameter (LVSD), and left ventricular ejection fraction (LVEF).

5.3 Follow-up arrangement

In this study, patient follow-up will be conducted by trained and fixed research staff who will be blinded

to the specific patient groups being assessed. Patients will be followed up every 4 weeks/1 month for a total of 52 weeks. During each visit, the number of missed days for heart failure treatment medications (such as beta-blockers, ACEI/ARB, mineralocorticoid receptor antagonists, recombinant human brain natriuretic peptide, angiotensin receptor-neprilysin inhibitors, etc.) will be recorded. Routine blood tests, liver and kidney function, electrolytes, blood lipids, NT-proBNP, and echocardiography will be performed every 4 weeks.

At the final follow-up visit, the NYHA functional class, Morisky Medication Adherence Scale, and MLHFQ score will be assessed. The proportion of days covered (PDC) for overall and each medication class will be calculated. Adverse events, unplanned cardiovascular hospitalizations, and all-cause mortality events will be recorded. The researchers will determine whether the adverse events are related to the treatment.

This approach ensures the objectivity and reliability of the study results by minimizing potential biases introduced by the assessment staff. The frequent follow-up visits and comprehensive data collection allow for a detailed analysis of medication adherence, treatment efficacy, and safety in patients with heart failure.

5.4 Study outcome measures

a. Primary efficacy endpoint: Overall Proportion of Days Covered (PDC) with heart failure medications at 52 weeks.

b. Secondary efficacy endpoints: Individual PDC for each class of treatment-related medications at 52 weeks; proportion of patients with overall and class-specific PDC ⩾ 80%; MLHFQ score at week 52; Morisky Medication Adherence Scale score; non-cardiovascular rehospitalization; all-cause mortality composite endpoint events; and days lost due to unplanned CV hospitalizations and death.

c. Safety endpoints: Number of adverse drug events. Common adverse events include hyperglycemia, electrolyte disturbances, sleep disorders, mood disorders, atrial fibrillation, ventricular tachycardia, acute coronary syndrome, infections, worsening renal function, dialysis, cardiogenic shock, and syncope.

6 Statistical analysis

6.1 Analytical purpose

A large-sample, multi-center, open-label, superiority randomized controlled trial will be conducted to compare the differences in medication adherence among chronic heart failure patients under pharmacist-led management versus conventional physician/nurse management. The study will also compare the effectiveness and safety of pharmacist-led management versus conventional physician/nurse management in the treatment of chronic heart failure.

6.2 Sample size selection

Based on previous research [16], the standard deviation of the overall proportion of days covered (PDC) for heart failure medications at 52 weeks is 14.9. Assuming a mean difference of 4.0 between the two groups, an alpha level of 0.05, and a beta level of 0.20, the calculated sample size for each group is 218. With an expected dropout rate of 10%, the sample size for each group is adjusted to 243, totaling 486 participants.

6.3 Analytical method

a. Statistical analysis was performed using SPSS 20.0 software.

b. The study utilized the full analysis set (patients who received at least one treatment and underwent at least one efficacy assessment) for efficacy analysis, and the safety set (patients who received at least one treatment and underwent at least one safety assessment) for safety analysis. Missing data were handled using the last observation carried forward (LOCF) method.

c. Continuous variables were described as mean ± standard deviation if normally distributed, or as median (interquartile range) if non-normally distributed. Categorical variables were presented as counts (percentages).

d. Between-group comparisons of continuous variables were conducted using independent samples t-test or Mann-Whitney U test. Categorical variables were compared using chi-square test or exact probability method.

e. Nelson-Aale survival curves were plotted for the composite endpoint of heart failure hospitalization and death. Group differences were assessed using the log-rank test.

f. Subgroup analyses will be conducted for the primary and secondary efficacy endpoints to assess the consistency of intervention effects across the following subgroups: age (<75 vs. ≥75 years), gender, heart rate at baseline (≤75 vs. >75 b.p.m.), NYHA class (I/II vs. III/IV), left ventricular ejection fraction (LVEF, <40% vs. ≥40%), history of diabetes, level of disease burden (number of different medications at baseline), baseline adherence, and quality of life at baseline.

g. statistical significance set at $P < 0.05$.

Reference

1. Chinese Medical Association, et al., Guidelines for the Diagnosis and Treatment of Chronic Heart Failure (2019). Chinese Journal of General Practice, 2019. 18(10): pp. 948-956.
2. Chinese Medical Association Cardiovascular Disease Branch Heart Failure Group, Chinese Physician Association Heart Failure Professional Committee, and Editorial Board of Chinese Journal of Cardiovascular Diseases, Chinese Guidelines for the Diagnosis and Treatment of Heart Failure 2018. Chinese Journal of Cardiovascular Diseases, 2018. 46(10): pp. 760-789.
3. National Center for Cardiovascular Diseases, Chinese Cardiovascular Disease Report 2018. 2019, Beijing, China: China Encyclopedia Press.
4. Zhang, Y., et al., *Contemporary Epidemiology, Management, and Outcomes of Patients Hospitalized for Heart Failure in China: Results From the China Heart Failure (China-HF) Registry.* J Card Fail, 2017. **23**(12): p. 868-875.
5. Pei Zhiyong, et al., A 15-Year Transition in Etiology and Short-Term Prognosis of Patients Hospitalized for Chronic Heart Failure. Chinese Journal of Cardiovascular Diseases, 2011. 39(5): pp. 434-439.

6. Liu Shaoshuai, et al., Analysis of the Clinical Features, Treatment Status, and Prognosis of Patients with Heart Failure in Different Grades of Hospitals in China. Chinese Journal of Molecular Cardiology, 2017. 017(002): pp. 2012-2016.
7. Chinese Medical Association Cardiovascular Disease Branch, Retrospective Survey of Hospitalized Cases of Chronic Heart Failure in Some Regions of China in 1980, 1990, and 2000. Chinese Journal of Cardiovascular Diseases, 2002. 30(8): pp. 450-454.
8. Zhou Haobin, et al., Retrospective Analysis of Clinical Characteristics and Prognosis in Patients with Different Ejection Fraction Heart Failure. Chinese Journal of Internal Medicine, 2017. 56(4): pp. 253-257.
9. Ponikowski, P., et al., *2016 ESC Guidelines for the diagnosis and treatment of acute and chronic heart failure: The Task Force for the diagnosis and treatment of acute and chronic heart failure of the European Society of Cardiology (ESC). Developed with the special contribution of the Heart Failure Association (HFA) of the ESC.* Eur J Heart Fail, 2016. **18**(8): p. 891-975.
10. Sun Lixing, et al., The Impact of Omaha System-Based Continuity Nursing Interventions on Medication Adherence in Outpatient Chronic Heart Failure Patients. Chinese Primary Health Care, 2016. 23(3): pp. 357-360.
11. Feng Jie, Huang Kang, and Lv Lian, Current Status of Readmission Rates and Related Influencing Factors in Patients with Chronic Heart Failure. Chinese Journal of Physicians, 2018. 20(08): pp. 1275-1278.
12. Schmidt, S., et al., *Acceptance of telemonitoring to enhance medication compliance in patients with chronic heart failure.* Telemed J E Health, 2008. **14**(5): p. 426-33.
13. Roblek, T., et al., *Clinical-pharmacist intervention reduces clinically relevant drug-drug interactions in patients with heart failure: A randomized, double-blind, controlled trial.* Int J Cardiol, 2016. **203**: p. 647-52.
14. Ewen, S., et al., *Analyses of drugs stored at home by elderly patients with chronic heart failure.* Clin Res Cardiol, 2015. **104**(4): p. 320-7.
15. Dempsey, J.T., et al., *Assessment of Drug Therapy-Related Issues in an Outpatient Heart Failure Population and the Potential Impact of Pharmacist-Driven Intervention.* J Pharm Pract, 2017. **30**(3): p. 318-323.
16. Schulz, M., et al., *Pharmacy-based interdisciplinary intervention for patients with chronic heart failure: results of the PHARM-CHF randomized controlled trial.* European journal of heart failure, 2019. **21**(8): p. 1012-1021.
17. Laufs, U., et al., *PHARMacy-based interdisciplinary program for patients with Chronic Heart Failure (PHARM-CHF): rationale and design of a randomized controlled trial, and results of the pilot study.* Eur J Heart Fail, 2018. **20**(9): p. 1350-1359.
18. Koshman, S.L., et al., *Pharmacist care of patients with heart failure: a systematic review of randomized trials.* Arch Intern Med, 2008. **168**(7): p. 687-94.
19. Roughead, E.E., et al., *The effectiveness of collaborative medicine reviews in delaying time to next hospitalization for patients with heart failure in the practice setting: results of a cohort study.* Circ Heart Fail, 2009. **2**(5): p. 424-8.
20. Pan Jin, et al., Evaluation of the Effectiveness of Clinical Pharmacists' Participation in the Clinical Pathway Management of Chronic Heart Failure. China Pharmacy, 2017. 28(23): pp. 3277-3281.
21. Chen Shuang, et al., Evaluation of the Effectiveness of Clinical Pharmacists' Participation in the

Disease Management of Chronic Heart Failure. China Pharmacy, 2016. 27(35): pp. 5021-5024.

22. Rao Lanlan and Ma Tianyi, Evaluation of the Effectiveness of Clinical Pharmacists' Participation in the Chronic Disease Management of Patients with Chronic Heart Failure. China Pharmacy, 2020. 31(06): pp. 745-750.
23. Huang Yuling, et al., Discussion on the Effectiveness of Clinical Pharmacists' Participation in the Disease Management of Chronic Heart Failure. Central South Pharmacy, 2016. 14(05): pp. 551-554.
24. Yu Feng, et al., The Impact of Pharmacist and Medical Staff Collaboration Management on Reducing Readmission Rates and Medical Expenses of Heart Failure Patients with Medical Insurance. Chinese Journal of Cardiology, 2017. 22(03): pp. 206-209.
25. Chen Tianjing, The Effect of Pharmacist Intervention on Improving Medication Adherence in Patients with Chronic Heart Failure. International Journal of Medicine and Health, 2019(14): pp. 2323-2325.
26. Dong, Y., et al., *Increased self-care activities and glycemic control rate in relation to health education via Wechat among diabetes patients: A randomized clinical trial.* Medicine (Baltimore), 2018. **97**(50): p. e13632.
27. Guo, Y., et al., *Development and Feasibility Testing of an mHealth (Text Message and WeChat) Intervention to Improve the Medication Adherence and Quality of Life of People Living with HIV in China: Pilot Randomized Controlled Trial.* JMIR Mhealth Uhealth, 2018. **6**(9): p. e10274.
28. He, C., et al., *Social Media-Promoted Weight Loss Among an Occupational Population: Cohort Study Using a WeChat Mobile Phone App-Based Campaign.* J Med Internet Res, 2017. **19**(10): p. e357.
29. Yang Xu and Yang Mo, The Role of WeChat in Continuity Follow-Up and Guidance for Patients with Heart Failure After Discharge. Journal of Changchun University of Chinese Medicine, 2016. 32(04): pp. 811-813.
30. Xie Chunyan, et al., The Application Effect of WeChat Group Nursing Intervention in the Home Rehabilitation of Patients with Chronic Heart Failure. General Nursing, 2017. 15(13): pp. 1651-1653.
31. Gao Jinxia, et al., Application of WeChat Platform in Continuity Nursing for Patients with Super-Elderly Chronic Heart Failure Complicated with Pulmonary Infection. Nursing Research, 2018. 32(23): pp. 3809-3811.
32. Zeng Qiuyun and Li Li, Study on the Influencing Factors of WeChat Follow-Up Compliance in Patients with Heart Valve Disease. Chinese Health Standard Management, 2016. 7(04): pp. 5-7.
33. World Medical, A., *World Medical Association Declaration of Helsinki: ethical principles for medical research involving human subjects.* JAMA, 2013. **310**(20): p. 2191-4.

Pharmacist-led management versus conventional physician/nurse management in medication adherence and efficacy among patients with chronic heart failure: A multicenter, randomized controlled study

Applicant Institution: The First Hospital of Hebei Medical University

Principal Investigator:Lingjiao Wang

Version 1.0

January 4, 2021

Researcher Consent Form

I agree to:

Take responsibility for the correct implementation of clinical research at our center.

Follow the research protocol, protocol amendments, and ethical committee requirements for conducting clinical trials.

Conduct the clinical study in compliance with Good Clinical Practice (GCP) guidelines and regulatory requirements.

Ensure that all personnel at our center involved in this study are fully acquainted with the study procedures, methods, investigational drugs involved, and their respective roles in the study.

Not modify or implement the research protocol without approval from the ethics committee, except when necessary to take urgent actions to minimize harm to subjects or for research management purposes (subject to approval by relevant regulatory authorities).

Researcher Signature / Date

____

Researcher Name (in block letters)

____

Research Institution

____

Contact Phone Number

____

1 Background

Chronic heart failure (CHF) is a complex clinical syndrome characterized by chronic structural and functional abnormalities of the heart. It represents a common outcome of acute and chronic cardiac conditions. Specifically, CHF refers to a persistent state of heart failure, typically presenting with symptoms such as dyspnea, fatigue, and fluid retention. The condition can exhibit a stable course, worsen over time, or become decompensated[1, 2]. According to reports, the prevalence of chronic heart failure (CHF) among adults in China ranges from 0.9% to 4.6%. As age increases, the incidence of CHF rises rapidly [3]. The main causes of heart failure in China include coronary heart disease, hypertension, dilated cardiomyopathy, valvular heart disease, and diabetes, among others [2-6]. Previous studies have shown that with the advancement of medical care in China, the in-hospital mortality rate of heart failure patients has significantly decreased [4, 6-8]. For instance, recent research indicates that the in-hospital mortality rate for hospitalized heart failure patients is approximately 4.1% [4], with a one-year mortality rate around 7.8%. However, the rate of heart failure readmissions is reported to be as high as 30% [6]. Therefore, as China's population ages and the prevalence of heart failure risk factors such as coronary heart disease, hypertension, and diabetes increases, coupled with improved medical care prolonging the survival of cardiac patients, the incidence of CHF is expected to continue rising [2, 3]. This trend will impose a significant burden on individuals and the healthcare system alike. Hence, urgent efforts are needed in the prevention and treatment of CHF.

Currently, drug therapy is the preferred method for treating CHF [1, 2, 9]. However, recent studies indicate that approximately 40% to 80% of CHF patients exhibit poor medication adherence, including under-dosing, missing doses, or discontinuing medications [10, 11]. Poor medication adherence is a significant factor leading to adverse outcomes in CHF patients, such as hospital readmissions [12]. In

addition, besides the medications recommended by guidelines, CHF patients often require additional drug treatments due to comorbidities and complications. This results in polypharmacy, increasing the risks of drug-drug interactions, adverse drug reactions, and poor adherence to medication [13-15]. Therefore, it is necessary to take measures to improve patient medication adherence and rationalize drug therapy to enhance treatment outcomes.

Several pharmacist-led or pharmacotherapy-based interventions for CHF patients conducted abroad [16-19] have shown that clinical pharmacists intervening in clinical settings can improve medication adherence, as well as enhance drug efficacy and safety. In China, a few studies have explored the impact of clinical pharmacist interventions on improving medication adherence, treatment outcomes, and quality of life in chronic heart failure patients, yielding conclusions similar to those observed in the aforementioned foreign studies [20-25]. In a randomized controlled trial [25], 100 chronic heart failure patients at risk of medication non-adherence were enrolled and randomly divided into two groups, each consisting of 50 patients. The control group received routine bedside and discharge medication guidance from doctors and nurses. In contrast, the intervention group received additional medication adherence interventions and guidance from clinical pharmacists at the hospital, in addition to routine medication instructions. The study compared medication adherence, clinical appropriateness of drug use, readmission rates, and mortality between the two groups. The results indicated that at 1 month and 3 months post-discharge, medication adherence in the intervention group was better than that in the control group (74% vs 52%; 94% vs 60.0%, respectively). Additionally, beyond 3 months post-discharge, the intervention group showed lower rates of clinically inappropriate drug use and readmission compared to the control group (14 cases vs 24 cases; 14% vs 32%). The previous domestic studies have the following limitations: mostly small sample sizes (less than 200 cases) and single-center studies; greater focus on

short-term adherence and safety efficacy indicators; clinical pharmacists often participate in related studies as participants rather than leaders. Therefore, it is necessary to conduct a multicenter, randomized controlled study with a large sample size to compare high-quality pharmacist-led management models with routine physician/nurse management models regarding long-term medication adherence and efficacy differences in patients with chronic heart failure.

It has been reported that WeChat has become China's most popular instant messaging application, with a monthly active user base reaching as high as 549 million [26]. WeChat's widespread adoption, timeliness, and convenience make it highly suitable for use in public health interventions in China. Numerous studies have already utilized WeChat for patient disease management, such as in diabetes [26], HIV/AIDS [27], demonstrating positive outcomes. Emerging social media platforms offer promising new methods for managing chronic health conditions. Recently, several studies have explored the continuous follow-up and guidance of heart failure patients using the WeChat platform [29-31]. The results indicate that these emerging WeChat follow-up methods help improve medication adherence among heart failure patients, promote clinically appropriate medication use, and enhance patient outcomes. In addition, establishing WeChat groups to disseminate relevant health education content about heart failure has been shown to guide discharged heart failure patients in home-based rehabilitation. This approach helps improve compliance with home-based rehabilitation and enhances patients' self-care abilities, thereby potentially improving patient outcomes [30]. However, there is currently a lack of research investigating the impact of pharmacist-led management models using WeChat to remind patients about medication adherence and provide patient education on the compliance and effectiveness outcomes for CHF (Congestive Heart Failure) patients. Additionally, existing studies in China on WeChat's involvement in disease management for heart failure patients often rely on single-center studies with small sample sizes.

Therefore, further validation through multicenter, large-sample, high-quality evidence is needed to substantiate these findings.

Based on the above, this study plans to conduct a multicenter, large-sample, open-label, pragmatic randomized controlled trial. Patients with chronic heart failure will be enrolled, and their baseline data will be collected. Stratification will be done based on preserved ejection fraction heart failure (HFpEF), reduced ejection fraction heart failure (HFrEF), and mid-range ejection fraction heart failure (HFmrEF). Participants will be randomly assigned to either a pharmacist-led intervention group or a conventional physician/nurse management group. Follow-up will be conducted for 52 weeks to compare the pharmacist-led intervention group with the conventional management group. Key outcomes will include medication possession ratio (PDC), incidence of mortality, and unplanned cardiovascular admissions. These measures aim to assess the impact of pharmacist-led intervention on medication adherence (non-pharmacological factors) and therapeutic efficacy (pharmacological factors) in chronic heart failure.

2 Study aims

(1) Comparison of differences in medication adherence among chronic heart failure patients under pharmacist-led management versus conventional physician/nurse management.

(2) Comparison of the effectiveness and safety of chronic heart failure treatment under pharmacist-led management versus conventional physician/nurse management.

3 Study participants

3.1 Inclusion criteria

All of the following entries are met:

A.Age $\geqslant$ 18 years old;

B.Diagnosed with chronic heart failure;

C.Stable on an established pharmacological treatment regimen for heart failure;

D.Subjects able to understand and sign the informed consent form.

3.2 Exclusion criteria

Exclclusion to any entry:

(1) Inability to comply with study-related assessments/follow-ups;

(2) Planned cardiac surgery during the study period;

(3) Life expectancy less than 1 year;

(4) History of malignant neoplasms;

(5) Severe hepatic or renal dysfunction (liver enzymes > 3 times the upper limit of normal or estimated glomerular filtration rate < 30 mL/min);

(6) Participation in another clinical study within the past three months or currently participating;

(7) Pregnant or lactating women.

3.3 Withdrawal Criteria

Exit upon meeting any entry:

(1)Occurrence of adverse events (including serious adverse events) deemed by the investigator as unsuitable to continue participation in the clinical study;

(2)Any situation where the investigator considers poor compliance;

(3)Loss to follow-up;

(4)Subject withdraws informed consent.

3.4 Discontinuation Criteria

Any entry is exclusion:

A.Diagnostic error where the subject does not meet the indications for this study. The subject is excluded

from the efficacy dataset but all safety observations are retained in the safety dataset.

B.Subjects who have been enrolled in the study but have not received any interventions.

Based on the above inclusion and exclusion criteria, patients were continuously recruited from the cardiology departments of the five hospitals: the First Hospital of Hebei Medical University, Hengshui People's Hospital, Tangshan Workers' Hospital and Tangshan Kailuan General Hospital.

The study was conducted in accordance with the Helsinki Declaration [33]. The study was approved by the ethics committees of the five hospitals mentioned above, and all participants signed informed consent forms.

4 Study design and treatment

4.1 Study design

The current project aims to conduct a large-sample, multicenter, open-label, superiority randomized controlled trial comparing the long-term medication adherence and treatment efficacy of pharmacist-led management versus conventional physician/nurse management for chronic heart failure patients. Patients with chronic heart failure will be enrolled, and their baseline data will be collected. They will be stratified based on heart failure with preserved ejection fraction (HFpEF), heart failure with reduced ejection fraction (HFrEF), and heart failure with mid-range ejection fraction (HFmrEF). Participants will be randomly divided into a pharmacist-led group and a traditional care group. The traditional care group will be managed by regular physicians and nurses, while the pharmacist-led group will receive pharmacist-directed interventions, utilizing WeChat-based patient management. After 52 weeks of follow-up, the medication possession ratio (PDC), mortality events, and unplanned cardiovascular hospitalizations will be compared between the two groups to evaluate the impact of pharmacist-led interventions on medication adherence (non-pharmacological factors) and treatment efficacy

(pharmacological factors) for chronic heart failure patients.

4.2 Randomization, grouping and blindness

Stratification based on LVEF: Patients will be stratified into three groups: heart failure with reduced ejection fraction (LVEF < 40%), heart failure with mid-range ejection fraction (LVEF ⩾ 40% and < 50%), and heart failure with preserved ejection fraction (LVEF ⩾ 50%). Using a computer-generated random number table, participants will be randomly assigned in a 1:1 ratio to the pharmacist-led group or the traditional care group, with stratification based on the LVEF levels. Opaque envelopes will be used for concealed allocation.

This study will be open-label for both researchers and patients, but blinded for the assessors.

4.3 Clinical Intervention Program

All interventions were initiated within 24h before hospital discharge.

Traditional mode group

a. Baseline:

The attending physician will order tests and examinations based on the patient's clinical presentation. Based on the results, adjustments will be made to the medication regimen, including medications for heart failure, hypertension, lipid regulation, and diabetes. A prescription for a 4-week/1-month supply of medication will be issued, and patients will be instructed to take their medication regularly.

The nurse will print out a medication list, distribute heart failure educational brochures, and provide a medication diary. Patients will be informed to record each medication taken in the diary. An appointment for the next follow-up visit (every 4 weeks/1 month) will be scheduled.

b.Subsequent Follow-up Visits:

The nurse will remind and confirm the appointment time seven days before each visit.

The nurse will check the patient's medication diary and compare it with the actual prescription.

The attending physician will order tests and examinations based on the patient's clinical presentation. Based on the results, adjustments will be made to the medication regimen, and a prescription for a 4-week supply of medication will be issued. Patients will be instructed to take their medication regularly.

The nurse will print out a medication list and remind the patient to record each medication taken in the diary. An appointment for the next follow-up visit will be scheduled.

During the follow-up process, if a patient is hospitalized, the physician will determine whether the hospitalization is related to heart failure or other adverse events during the subsequent visit. The follow-up visit will then continue according to the aforementioned process.

Pharmacist-Led group

a. Baseline:

Here is the translation of the Chinese text you sent into English:

The pharmacist will inspect all medication packages to understand the patient's actual adherence to the medication regimen and compare it with the prescription.

The attending physician will order tests and examinations based on the patient's clinical presentation. The physician will then discuss with the pharmacist regarding any necessary adjustments to the medication regimen, including medications for heart failure, hypertension, lipid regulation, and diabetes.

Based on the discussion, the physician will issue a prescription for a 4-week/1-month supply of medication.

The pharmacist will conduct patient education, explaining the treatment goals for each condition, the role and necessity of the medications, dosage and administration instructions, possible adverse events, management strategies, and drug-drug interactions.

The pharmacist will use medication boxes to dispense the medications, label them with dosage and administration instructions, print out the medication list, distribute heart failure educational brochures, and provide a medication diary. Patients will be instructed to record each medication taken in the diary. An appointment for the next follow-up visit (every 4 weeks/1 month) will be scheduled.

The pharmacist will add the patient or their caregiver as a WeChat friend and create a patient group chat. The pharmacist will privately remind the patient once a week to take their medication and record it in the medication diary. If the patient or their caregiver needs medication or disease-related advice, they can privately message the pharmacist or use the group chat, and the pharmacist will provide a response.

b.Subsequent Follow-up Visits:

Seven days before the visit, the pharmacist will remind and confirm the appointment time.

During the visit, the pharmacist will check the patient's medication diary, compare it with the actual prescription, and inquire about any adverse events that occurred or any new medications taken.

The attending physician will order tests and examinations based on the patient's clinical presentation and discuss with the pharmacist any necessary adjustments to the medication regimen.

The physician will issue a prescription for a 4-week/1-month supply of medication.

The pharmacist will conduct patient education based on the medication adherence in the past 4 weeks/1 month, focusing on any incorrectly taken, missed, newly added, or adjusted medications. This education will cover the role, necessity, dosage and administration instructions, possible adverse events, management strategies, and drug-drug interactions of the medications.

The pharmacist will use medication boxes to dispense the medications, label them with dosage and administration instructions, and instruct the patient to record each medication taken in the diary. An appointment for the next follow-up visit will be scheduled.

During the follow-up process, if a patient is hospitalized, the physician will determine whether the hospitalization is related to heart failure or other adverse events during the subsequent visit. The follow-up visit will then continue according to the aforementioned process.

5 Study planning and data collection

The original data to be collected for this study originates from the original medical records filled in by the researchers and the original records filled in by the research subjects. The researchers are required to guide the research subjects in accordance with the study protocol for the prescribed treatment, conduct follow-up visits at the scheduled times, and promptly organize and save all relevant records or results. The research subjects must fill in the corresponding data in a timely and detailed manner as requested by the researchers and promptly submit them for preservation.

5.1 Laboratory examination

Blood routine test, liver function, kidney function, electrolytes, glycosylated hemoglobin (HbA1c), blood lipids, N-terminal pro-B-type natriuretic peptide (NT-proBNP)/B-type natriuretic peptide (BNP), etc.

5.2 Baseline data and indicators

Demographic data, past medical history, smoking and alcohol history, vital signs, time of heart failure diagnosis, etiology, current treatment medications, Minnesota Living with Heart Failure Questionnaire (MLHFQ) score, Morisky Medication Adherence Scale, New York Heart Association (NYHA) functional class, blood routine test, liver and kidney function, electrolytes, fasting blood glucose, glycosylated hemoglobin (HbA1c), blood lipids, NT-proBNP/BNP, chest X-ray, echocardiography including left ventricular end-diastolic diameter (LVDd), left ventricular end-systolic diameter (LVSD), and left ventricular ejection fraction (LVEF).

5.3 Follow-up arrangement

In this study, patient follow-up will be conducted by trained and fixed research staff who will be blinded to the specific patient groups being assessed. Patients will be followed up every 4 weeks/1 month for a total of 52 weeks. During each visit, the number of missed days for heart failure treatment medications (such as beta-blockers, ACEI/ARB, mineralocorticoid receptor antagonists, recombinant human brain natriuretic peptide, angiotensin receptor-neprilysin inhibitors, etc.) will be recorded. Routine blood tests, liver and kidney function, electrolytes, blood lipids, NT-proBNP, and echocardiography will be performed every 4 weeks.

At the final follow-up visit, the NYHA functional class, Morisky Medication Adherence Scale, and MLHFQ score will be assessed. The proportion of days covered (PDC) for overall and each medication class will be calculated. Adverse events, unplanned cardiovascular hospitalizations, and all-cause mortality events will be recorded. The researchers will determine whether the adverse events are related to the treatment.

This approach ensures the objectivity and reliability of the study results by minimizing potential biases introduced by the assessment staff. The frequent follow-up visits and comprehensive data collection allow for a detailed analysis of medication adherence, treatment efficacy, and safety in patients with heart failure.

5.4 Study outcome measures

a. Primary efficacy endpoint: Overall Proportion of Days Covered (PDC) with heart failure medications at 52 weeks.

b. Secondary efficacy endpoints: Individual PDC for each class of treatment-related medications at 52 weeks; proportion of patients with overall and class-specific PDC ≥ 80%; NYHA functional

classification at week 52; MLHFQ score at week 52; NT-proBNP/BNP levels; differences in cardiac function indicators (e.g., LVDd, LVSD, LVEF) from baseline; non-cardiovascular rehospitalization; all-cause mortality composite endpoint events.

c. Safety endpoints: Number and frequency of adverse drug events. Common adverse events include hyperglycemia, electrolyte disturbances, sleep disorders, mood disorders, atrial fibrillation, ventricular tachycardia, acute coronary syndrome, infections, worsening renal function, dialysis, cardiogenic shock, and syncope.

6 Statistical analysis

6.1 Analytical purpose

A large-sample, multi-center, open-label, superiority randomized controlled trial will be conducted to compare the differences in medication adherence among chronic heart failure patients under pharmacist-led management versus conventional physician/nurse management. The study will also compare the effectiveness and safety of pharmacist-led management versus conventional physician/nurse management in the treatment of chronic heart failure.

6.2 Sample size selection

Based on previous research [16], the standard deviation of the overall proportion of days covered (PDC) for heart failure medications at 52 weeks is 14.9. Assuming a mean difference of 4.0 between the two groups, an alpha level of 0.05, and a beta level of 0.20, the calculated sample size for each group is 218. With an expected dropout rate of 10%, the sample size for each group is adjusted to 243, totaling 486 participants.

6.3 Analytical method

a. Statistical analysis was performed using SPSS 20.0 software.

b. The study utilized the full analysis set (patients who received at least one treatment and underwent at least one efficacy assessment) for efficacy analysis, and the safety set (patients who received at least one treatment and underwent at least one safety assessment) for safety analysis. Missing data were handled using the last observation carried forward (LOCF) method.

c. Continuous variables were described as mean ± standard deviation if normally distributed, or as median (interquartile range) if non-normally distributed. Categorical variables were presented as counts (percentages).

d. Between-group comparisons of continuous variables were conducted using independent samples t-test or Mann-Whitney U test. Categorical variables were compared using chi-square test or exact probability method.

e. Nelson-Aale survival curves were plotted for the composite endpoint of heart failure hospitalization and death. Group differences were assessed using the log-rank test.

f. statistical significance set at $P < 0.05$.

Reference

1. Chinese Medical Association, et al., Guidelines for the Diagnosis and Treatment of Chronic Heart Failure (2019). Chinese Journal of General Practice, 2019. 18(10): pp. 948-956.
2. Chinese Medical Association Cardiovascular Disease Branch Heart Failure Group, Chinese Physician Association Heart Failure Professional Committee, and Editorial Board of Chinese Journal of Cardiovascular Diseases, Chinese Guidelines for the Diagnosis and Treatment of Heart Failure 2018. Chinese Journal of Cardiovascular Diseases, 2018. 46(10): pp. 760-789.
3. National Center for Cardiovascular Diseases, Chinese Cardiovascular Disease Report 2018. 2019, Beijing, China: China Encyclopedia Press.
4. Zhang, Y., et al., *Contemporary Epidemiology, Management, and Outcomes of Patients Hospitalized for Heart Failure in China: Results From the China Heart Failure (China-HF) Registry.* J Card Fail, 2017. **23**(12): p. 868-875.
5. Pei Zhiyong, et al., A 15-Year Transition in Etiology and Short-Term Prognosis of Patients Hospitalized for Chronic Heart Failure. Chinese Journal of Cardiovascular Diseases, 2011. 39(5): pp. 434-439.
6. Liu Shaoshuai, et al., Analysis of the Clinical Features, Treatment Status, and Prognosis of

Patients with Heart Failure in Different Grades of Hospitals in China. Chinese Journal of Molecular Cardiology, 2017. 017(002): pp. 2012-2016.
7. Chinese Medical Association Cardiovascular Disease Branch, Retrospective Survey of Hospitalized Cases of Chronic Heart Failure in Some Regions of China in 1980, 1990, and 2000. Chinese Journal of Cardiovascular Diseases, 2002. 30(8): pp. 450-454.
8. Zhou Haobin, et al., Retrospective Analysis of Clinical Characteristics and Prognosis in Patients with Different Ejection Fraction Heart Failure. Chinese Journal of Internal Medicine, 2017. 56(4): pp. 253-257.
9. Ponikowski, P., et al., *2016 ESC Guidelines for the diagnosis and treatment of acute and chronic heart failure: The Task Force for the diagnosis and treatment of acute and chronic heart failure of the European Society of Cardiology (ESC). Developed with the special contribution of the Heart Failure Association (HFA) of the ESC.* Eur J Heart Fail, 2016. **18**(8): p. 891-975.
10. Sun Lixing, et al., The Impact of Omaha System-Based Continuity Nursing Interventions on Medication Adherence in Outpatient Chronic Heart Failure Patients. Chinese Primary Health Care, 2016. 23(3): pp. 357-360.
11. Feng Jie, Huang Kang, and Lv Lian, Current Status of Readmission Rates and Related Influencing Factors in Patients with Chronic Heart Failure. Chinese Journal of Physicians, 2018. 20(08): pp. 1275-1278.
12. Schmidt, S., et al., *Acceptance of telemonitoring to enhance medication compliance in patients with chronic heart failure.* Telemed J E Health, 2008. **14**(5): p. 426-33.
13. Roblek, T., et al., *Clinical-pharmacist intervention reduces clinically relevant drug-drug interactions in patients with heart failure: A randomized, double-blind, controlled trial.* Int J Cardiol, 2016. **203**: p. 647-52.
14. Ewen, S., et al., *Analyses of drugs stored at home by elderly patients with chronic heart failure.* Clin Res Cardiol, 2015. **104**(4): p. 320-7.
15. Dempsey, J.T., et al., *Assessment of Drug Therapy-Related Issues in an Outpatient Heart Failure Population and the Potential Impact of Pharmacist-Driven Intervention.* J Pharm Pract, 2017. **30**(3): p. 318-323.
16. Schulz, M., et al., *Pharmacy-based interdisciplinary intervention for patients with chronic heart failure: results of the PHARM-CHF randomized controlled trial.* European journal of heart failure, 2019. **21**(8): p. 1012-1021.
17. Laufs, U., et al., *PHARMacy-based interdisciplinary program for patients with Chronic Heart Failure (PHARM-CHF): rationale and design of a randomized controlled trial, and results of the pilot study.* Eur J Heart Fail, 2018. **20**(9): p. 1350-1359.
18. Koshman, S.L., et al., *Pharmacist care of patients with heart failure: a systematic review of randomized trials.* Arch Intern Med, 2008. **168**(7): p. 687-94.
19. Roughead, E.E., et al., *The effectiveness of collaborative medicine reviews in delaying time to next hospitalization for patients with heart failure in the practice setting: results of a cohort study.* Circ Heart Fail, 2009. **2**(5): p. 424-8.
20. Pan Jin, et al., Evaluation of the Effectiveness of Clinical Pharmacists' Participation in the Clinical Pathway Management of Chronic Heart Failure. China Pharmacy, 2017. 28(23): pp. 3277-3281.
21. Chen Shuang, et al., Evaluation of the Effectiveness of Clinical Pharmacists' Participation in the Disease Management of Chronic Heart Failure. China Pharmacy, 2016. 27(35): pp. 5021-5024.

22. Rao Lanlan and Ma Tianyi, Evaluation of the Effectiveness of Clinical Pharmacists' Participation in the Chronic Disease Management of Patients with Chronic Heart Failure. China Pharmacy, 2020. 31(06): pp. 745-750.
23. Huang Yuling, et al., Discussion on the Effectiveness of Clinical Pharmacists' Participation in the Disease Management of Chronic Heart Failure. Central South Pharmacy, 2016. 14(05): pp. 551-554.
24. Yu Feng, et al., The Impact of Pharmacist and Medical Staff Collaboration Management on Reducing Readmission Rates and Medical Expenses of Heart Failure Patients with Medical Insurance. Chinese Journal of Cardiology, 2017. 22(03): pp. 206-209.
25. Chen Tianjing, The Effect of Pharmacist Intervention on Improving Medication Adherence in Patients with Chronic Heart Failure. International Journal of Medicine and Health, 2019(14): pp. 2323-2325.
26. Dong, Y., et al., *Increased self-care activities and glycemic control rate in relation to health education via Wechat among diabetes patients: A randomized clinical trial.* Medicine (Baltimore), 2018. **97**(50): p. e13632.
27. Guo, Y., et al., *Development and Feasibility Testing of an mHealth (Text Message and WeChat) Intervention to Improve the Medication Adherence and Quality of Life of People Living with HIV in China: Pilot Randomized Controlled Trial.* JMIR Mhealth Uhealth, 2018. **6**(9): p. e10274.
28. He, C., et al., *Social Media-Promoted Weight Loss Among an Occupational Population: Cohort Study Using a WeChat Mobile Phone App-Based Campaign.* J Med Internet Res, 2017. **19**(10): p. e357.
29. Yang Xu and Yang Mo, The Role of WeChat in Continuity Follow-Up and Guidance for Patients with Heart Failure After Discharge. Journal of Changchun University of Chinese Medicine, 2016. 32(04): pp. 811-813.
30. Xie Chunyan, et al., The Application Effect of WeChat Group Nursing Intervention in the Home Rehabilitation of Patients with Chronic Heart Failure. General Nursing, 2017. 15(13): pp. 1651-1653.
31. Gao Jinxia, et al., Application of WeChat Platform in Continuity Nursing for Patients with Super-Elderly Chronic Heart Failure Complicated with Pulmonary Infection. Nursing Research, 2018. 32(23): pp. 3809-3811.
32. Zeng Qiuyun and Li Li, Study on the Influencing Factors of WeChat Follow-Up Compliance in Patients with Heart Valve Disease. Chinese Health Standard Management, 2016. 7(04): pp. 5-7.
33. World Medical, A., *World Medical Association Declaration of Helsinki: ethical principles for medical research involving human subjects.* JAMA, 2013. **310**(20): p. 2191-4.

Title:Pharmacist-led management versus conventional physician/nurse management in medication adherence and efficacy among patients with chronic heart failure: A multicenter, randomized controlled study

Statistical Analysis Plan

Version 1.0

11 November, 2021

Principal	Chunhua Zhou

Investigator:	Affiliations: Department of Clinical Pharmacy, The First Hospital of Hebei Medical University, No.89 Donggang St, Yuhua District, Hebei 050000, China.<br>Lingjiao Wang (The First Hospital of Hebei Medical University); Baogui Chong (Hengshui People's Hospital); Liping Han (Handan First Hospital); Huan Zhang (Cangzhou Central Hospital); Aixia Liu (The Fourth Hospital of Handan).
Methodologist and statistician:	Chunhua Zhou<br>Affiliations: Department of Clinical Pharmacy, The First Hospital of Hebei Medical University, No.89 Donggang St, Yuhua District, Hebei 050000, China.<br><br>Data analyst:<br>Jing Zhao (The Second Hospital of Hebei Medical University)
Inclusion and exclusion criteria	Inclusion Criteria (all must be met):<br>1.Age ⩾ 18 years old;<br>2.Diagnosed with chronic heart failure;<br>3.Stable on an established pharmacological treatment regimen for heart failure;<br>4.Subjects able to understand and sign the informed consent form.<br>Exclusion Criteria (any of the following):<br>1.Inability to comply with study-related assessments/follow-ups;<br>2.Planned cardiac surgery during the study period;<br>3.Life expectancy less than 1 year;<br>4.History of malignant neoplasms;<br>5.Severe hepatic or renal dysfunction (liver enzymes > 3 times the upper limit of normal or estimated glomerular filtration rate < 30 mL/min);<br>6.Participation in another clinical study within the past three months or currently participating;<br>7.Pregnant or lactating women.<br>Withdrawal Criteria (any of the following):<br>1.Occurrence of adverse events (including serious adverse events) deemed by the investigator as unsuitable to continue participation in the clinical study;<br>2.Any situation where the investigator considers poor compliance;<br>3.Loss to follow-up;<br>4.Subject withdraws informed consent.<br>Discontinuation Criteria (any of the following):<br>1.Diagnostic error where the subject does not meet the indications for this study. The subject is excluded from the efficacy dataset but all safety observations are retained in the safety dataset.<br>2.Subjects who have been enrolled in the study but have not received any interventions.

Randomization and treatment procedures	Based on LVEF stratification: heart failure with reduced ejection fraction (LVEF < 40%), heart failure with mid-range ejection fraction (LVEF ≥ 40% and < 50%), and heart failure with preserved ejection fraction (LVEF ≥ 50%), totaling 3 strata. Using a computer-generated random number table, subjects are randomized 1:1 into a pharmacist-led group and a traditional care group using opaque envelopes for concealed allocation. Blinding is not implemented for researchers and patients in this study, but the assessors are blinded. All interventions commence within 24 hours prior to discharge.<br>In this study, patient follow-up will be conducted by trained and designated study personnel. Blinding of assessors will be implemented, where the assessors will not know the specific group assignments of the patients they are evaluating.<br>Patients will be followed up every 4 weeks for a total of 52 weeks in this study. The follow-up will involve recording the number of missed days for heart failure treatment medications (beta-blockers, ACEI/ARB, mineralocorticoid receptor antagonists, diuretics, etc.). Every 4 weeks, blood tests including complete blood count, liver and kidney function tests, electrolytes, lipid profile, NT-proBNP/BNP levels, and echocardiography will be conducted.<br>At the final follow-up, assessments will include NYHA functional classification, Morisky Medication Adherence Scale, and Minnesota Living with Heart Failure Questionnaire (MLHFQ). The overall and individual medication class-specific Proportion of Days Covered (PDC) will be calculated. Adverse events, non-cardiovascular rehospitalizations, and all-cause mortality events will be recorded, with the researcher determining whether adverse events are treatment-related.
Outcome	Primary efficacy endpoint: Overall Proportion of Days Covered (PDC) with heart failure medications at 52 weeks.<br>Secondary efficacy endpoints: Individual PDC for each class of treatment-related medications at 52 weeks; proportion of patients with overall and class-specific PDC ≥ 80%; MLHFQ score at week 52; Morisky Medication Adherence Scale score; non-cardiovascular rehospitalization; all-cause mortality composite endpoint events, and days lost due to unplanned CV hospitalizations and death.<br>Safety endpoints: Number of adverse drug events. Common adverse events include hyperglycemia, electrolyte disturbances, sleep disorders, mood disorders, atrial fibrillation, ventricular tachycardia, acute coronary syndrome, infections, worsening renal function, dialysis, cardiogenic shock, and syncope.
Statistical analysis methods	1. Statistical analysis was performed using SPSS 20.0 software.<br>2. The study utilized the full analysis set (patients who received at least one treatment and underwent at least one efficacy assessment) for efficacy analysis, and the safety set (patients who received at least one treatment and

	underwent at least one safety assessment) for safety analysis. Missing data were handled using the last observation carried forward (LOCF) method.<br>3. Continuous variables were described as mean ± standard deviation if normally distributed, or as median (interquartile range) if non-normally distributed. Categorical variables were presented as counts (percentages).<br>4. Between-group comparisons of continuous variables were conducted using independent samples t-test or Mann-Whitney U test. Categorical variables were compared using chi-square test or exact probability method.<br>5. Nelson-Aale survival curves were plotted for the composite endpoint of heart failure hospitalization and death. Group differences were assessed using the log-rank test.<br>6. Subgroup analyses will be conducted for the primary and secondary efficacy endpoints to assess the consistency of intervention effects across the following subgroups: age (<75 vs. ≥75 years), gender, heart rate at baseline (≤75 vs. >75 b.p.m.), NYHA class (I/II vs. III/IV), left ventricular ejection fraction (LVEF, <40% vs. ≥40%), history of diabetes, level of disease burden (number of different medications at baseline), baseline adherence, and quality of life at baseline.<br>7. statistical significance set at $P < 0.05$.

1. Background

Chronic heart failure (CHF) is a complex clinical syndrome characterized by chronic structural and functional abnormalities of the heart. Currently, drug therapy is the preferred method for treating CHF. However, recent studies indicate that approximately 40% to 80% of CHF patients exhibit poor medication adherence, including under-dosing, missing doses, or discontinuing medications. Poor medication adherence is a significant factor leading to adverse outcomes in CHF patients, such as hospital readmissions. In addition, besides the medications recommended by guidelines, CHF patients often require additional drug treatments due to comorbidities and complications. This results in polypharmacy, increasing the risks of drug-drug interactions, adverse drug reactions, and poor adherence to medication. Therefore, it is necessary to take measures to improve patient medication adherence and rationalize drug therapy to enhance treatment outcomes.

Based on the above, this study plans to conduct a multicenter, large-sample, open-label, pragmatic randomized controlled trial. Patients with chronic heart failure will be enrolled, and their baseline

data will be collected. Stratification will be done based on preserved ejection fraction heart failure (HFpEF), reduced ejection fraction heart failure (HFrEF), and mid-range ejection fraction heart failure (HFmrEF). Participants will be randomly assigned to either a pharmacist-led intervention group or a conventional physician/nurse management group. Follow-up will be conducted for 52 weeks to compare the pharmacist-led intervention group with the conventional management group. Key outcomes will include medication possession ratio (PDC), incidence of mortality, and unplanned cardiovascular admissions. These measures aim to assess the impact of pharmacist-led intervention on medication adherence (non-pharmacological factors) and therapeutic efficacy (pharmacological factors) in chronic heart failure.

2. Study aims

(1) Comparison of differences in medication adherence among chronic heart failure patients under pharmacist-led management versus conventional physician/nurse management.

(2) Comparison of the effectiveness and safety of chronic heart failure treatment under pharmacist-led management versus conventional physician/nurse management.

3. Hypothesis

Compared with the traditional group, the medication adherence of the pharmacist management model group increased by 5% after 12 months of follow-up.

4. Study Design: Multicenter, Randomized Controlled Study

5. Analysis population

5.1 Flow diagram

Please see manuscript Figure 1.

5.2 Definition of the analysis population

CHF is defined based on the following criteria:

(1) The presence of symptoms and signs of heart failure, such as difficulty breathing, fatigue, and fluid retention.

(2) Abnormal cardiac structure and/or function as determined by echocardiography.

(3) Elevated levels of natriuretic peptides, such as B-type Natriuretic Peptide (BNP) > 35 ng/L and/or N-terminal proBNP (NT-proBNP) > 125 ng/L.

Stable heart failure medication regimen: patients have not experienced an acute exacerbation of heart failure—such as a sudden worsening of heart failure symptoms and signs—nor have they required any adjustments to their heart failure medication in the week prior to enrollment in the study.

Inclusion Criteria (all must be met):

(1) Age ≥ 18 years old;

(2) Diagnosed with chronic heart failure;

(3) Stable on an established pharmacological treatment regimen for heart failure;

(4) Subjects able to understand and sign the informed consent form.

Exclusion Criteria (any of the following):

(1) Inability to comply with study-related assessments/follow-ups;

(2) Planned cardiac surgery during the study period;

(3) Life expectancy less than 1 year;

(4) History of malignant neoplasms;

(5) Severe hepatic or renal dysfunction (liver enzymes > 3 times the upper limit of normal or estimated glomerular filtration rate < 30 mL/min);

(6) Participation in another clinical study within the past three months or currently participating;

(7) Pregnant or lactating women.

Withdrawal Criteria (any of the following):

(1) Occurrence of adverse events (including serious adverse events) deemed by the investigator as unsuitable to continue participation in the clinical study;

(2) Any situation where the investigator considers poor compliance;

(3) Loss to follow-up;

(4) Subject withdraws informed consent.

Discontinuation Criteria (any of the following):

(1) Diagnostic error where the subject does not meet the indications for this study. The subject is excluded from the efficacy dataset but all safety observations are retained in the safety dataset.

(2) Subjects who have been enrolled in the study but have not received any interventions.

5.3 Sample size

Based on previous studies (Schulz, M., et al. European Journal of Heart Failure, 2019), the overall standard deviation of PDC for heart failure medication at 52 weeks is 14.9. Assuming a mean difference of 4.0 between the two groups, with α set at 0.05 and β at 0.20, each group would require 218 participants, accounting for an expected attrition rate of 10%. Thus, each group would need 243 participants, totaling 486 participants overall.

6. Outcomes

Primary efficacy endpoint:

Overall Proportion of Days Covered (PDC) with heart failure medications at 52 weeks.

Secondary efficacy endpoints:

Individual PDC for each class of treatment-related medications at 52 weeks; proportion of patients with overall and class-specific PDC ≥ 80%; MLHFQ score at week 52; Morisky Medication Adherence Scale score; non-cardiovascular rehospitalization; all-cause mortality composite endpoint events, and days lost due to unplanned CV hospitalizations and death.

Safety endpoints: Number of adverse drug events. Common adverse events include hyperglycemia, electrolyte disturbances, sleep disorders, mood disorders, atrial fibrillation, ventricular tachycardia, acute coronary syndrome, infections, worsening renal function, dialysis, cardiogenic shock, and syncope.

7. Statistical Analysis

7.1 Descriptive analyses

Table 1 displays the descriptive characteristics of the control group and the intervention group. We collected demographic data, smoking and alcohol history, vital signs, comorbidities, current medications, MLHFQ scores, Morisky Medication Adherence Scale, New York Heart Association (NYHA) functional class, laboratory routine tests for liver and kidney function, electrolytes, and echocardiograms for both groups. Continuous variables are described as mean ± standard deviation if normally distributed, and as median (interquartile range) if not normally distributed. Categorical variables are expressed as counts (percentages). To assess the balance between the control and intervention groups after randomization, we descriptively compared the baseline characteristics using two-sample t-tests, χ^2 tests, or Wilcoxon rank-sum tests.

7.2 Primary and Secondary efficacy outcomes analyses

Table 2 and Supplement 2 in the manuscript presents the primary and secondary outcomes. Figure 2 displays the composite endpoint of hospitalization for heart failure and death.

We compared the baseline and 12-month follow-up data changes between the control group and the intervention group. The data collection process strictly adhered to privacy protection and data security regulations and standards. We conducted efficacy analysis using the full analysis set (patients who received at least one treatment and underwent at least one efficacy assessment) and safety analysis using the safety set (patients who received at least one treatment and underwent at least one safety assessment).

For each patient, we calculated the number of days they had a supply of heart failure medication during the 52-week follow-up period. We made adjustments based on the patient's hospitalization days, medication switches, and death. For hospitalization days, we assumed that the patient did not deplete their medication supply during these days, so these days were excluded from the calculation. If a patient switched medications within a therapeutic category, the patient's medication supply was replaced by the new supply. If a patient died during the follow-up period, all days after death were excluded. We calculated the adherence for each medication category, ranging from 0 to 1.0, and then averaged across all non-missing medication categories to derive the summary PDC. Continuous variables were compared between groups using independent sample t-tests or Wilcoxon rank-sum tests. Categorical variables were compared using chi-square tests. We performed multiple testing corrections for all secondary endpoints. We employed the Bonferroni correction method to control the family-wise error rate (FWER), ensuring the reliability of the results. We plotted the Nelson-Aalen survival curves for the composite endpoint of heart failure hospitalization and death. The log-rank test was used to assess differences between groups.

7.3 Missing data

We assessed the distribution and degree of missingness for all variables. Outliers or inconsistent

data will be discussed by consensus among the survey teams. Missing data will be handled using the Last Observation Carried Forward (LOCF) method.

7.4 Trial monitoring

To ensure the quality and consistency of data across centers, we conducted standardized training for pharmacists involved in the study according to the standards of pharmaceutical services in China and the requirements of the study. Other personnel were also trained according to the study's requirements.

At the outset of the project, we established a Data Management Committee composed of experts in the fields of pharmacy, clinical practice, and statistics. The committee conducted quality control of data related to adverse drug events, death cases, and pharmacist interventions at the midpoint and conclusion of the trial.

7.5 Subgroup analyses

To assess the impact of potential factors on the outcomes, we conducted subgroup analyses for the primary and secondary efficacy endpoints to evaluate the consistency of the intervention effects across the following subgroups: age (<75 vs. ≥75 years), gender, baseline heart rate (≤75 vs. >75 bpm), NYHA class (I/II vs. III/IV), left ventricular ejection fraction (LVEF, <40% vs. ≥40%), history of diabetes, disease burden level (number of different medications used at baseline), baseline adherence, and quality of life at baseline. The results of the subgroup analyses were presented using forest plots.

7.6 Safety outcomes analysis

The safety outcome is the number of adverse drug events (ADEs) identified within the 52-week follow-up period. We used the chi-squared test to calculate the safety results. In our study, ADEs

were defined as "harmful reactions unrelated to the intended medicinal purpose that occur with the normal use of a qualified drug."
