# Supplementary material for: Pharmacist-Led Management Model and Medication Adherence Among Patients With Chronic Heart Failure: A Randomized Clinical Trial
Source: JAMA Netw Open. 2024 Dec 20;7(12):e2453976. doi: 10.1001/jamanetworkopen.2024.53976 (PMC11662253; doi:10.1001/jamanetworkopen.2024.53976)
Supplement: Supplement 2. — eTable 1. The Outcomes of Morisky Scores and MLHFQ Scores eTable 2. Safety Outcomes eTable 3. All-Cause Deaths and Unplanned Cardiovascular Hospitalizations During 52-Week Follow-Up [file jamanetwopen-e2453976-s002.pdf]

Supplemental Online Content

Wang L, Zhao Y, Han L, et al. Pharmacist-led management model and medication adherence among patients with chronic heart failure: a randomized clinical trial. *JAMA Netw Open*. 2024;7(12):e2453976. doi:10.1001/jamanetworkopen.2024.53976

eTable 1. The Outcomes of Morisky Scores and MLHFQ Scores
eTable 2. Safety Outcomes
eTable 3. All-Cause Deaths and Unplanned Cardiovascular Hospitalizations During 52-Week Follow-Up

This supplemental material has been provided by the authors to give readers additional information about their work.

© 2024 Wang L et al. *JAMA Network Open*.

eTable 1. The outcomes of Morisky scores and MLHFQ scores

Outcome	Intervention group (n = 223)	Control group (n =222)	*P* value
Morisky scores，Median (IQR)	7.8 (6.8-8.0)	7.3 (4.5-7.8)	0.001
MLHFQ scores，Median (IQR)	25.0 (12.0-40.0)	35.0 (20.0-44.0)	0.005

Abbreviations:
MLHFQ, Minnesota Living with Heart Failure Questionnaire; IQR, interquartile range.

eTable 2. Safety outcomes

NO.	Intervention group (n = 223)	Control group (n =222)	*P* value
AEs	19	15	
No AE	204	207	0.48

Abbreviations:AEs, adverse events.

eTable 3. All-cause deaths and unplanned cardiovascular hospitalizations during 52-week follow-up

Events	Intervention group (n = 223)	Control group (n =222)	*P* value
All-cause deaths, n	4	7	0.36
Unplanned CV hospitalizations, No.%	59 (26.5)	65 (29.2)	0.51
Days lost due to unplanned CV hospitalizations and death, mean (95% CI)	27.3 (13.6-40.9)	33.6 (15.8-51.5)	0.58

Abbreviations: CI, confidence interval.

© 2024 Wang L et al. *JAMA Network Open*.
