# Supplementary material for: Pharmacist-Led Management Model and Medication Adherence Among Patients With Chronic Heart Failure: A Randomized Clinical Trial
Source: JAMA Netw Open. 2024 Dec 20;7(12):e2453976. doi: 10.1001/jamanetworkopen.2024.53976 (PMC11662253; doi:10.1001/jamanetworkopen.2024.53976)
Supplement: Supplement 3. — Data Sharing Statement [file jamanetwopen-e2453976-s003.pdf]

Data Sharing Statement

Wang. Pharmacist-Led Management Model and Medication Adherence Among Patients With Chronic Heart Failure. *JAMA Netw Open*. Published December 20, 2024.
doi:10.1001/jamanetworkopen.2024.53976

Data
Additional Information: Chinese Clinical Trial Registry Identifier: ChiCTR2000040232
Data available: Yes
Data types: Deidentified participant data
How to access data: zhouchunhua@hebmu.edu.cn
When available: With publication

Supporting Documents
Document types: Informed consent form
How to access documents: zhouchunhua@hebmu.edu.cn
When available: With publication

Additional Information
Who can access the data: researchers whose proposed use of the data has been approved
Types of analyses: for a specified purpose
Mechanisms of data availability: with a signed data access agreement
Any additional restrictions: no
